# Supplementary material for: Synthesis and analysis of 4-(3-fluoropropyl)-glutamic acid stereoisomers to determine the stereochemical purity of (4S)-4-(3-[18F]fluoropropyl)-L-glutamic acid ([18F]FSPG) for clinical use
Source: PLoS One. 2020 Dec 14;15(12):e0243831. doi: 10.1371/journal.pone.0243831 (PMC7735610; doi:10.1371/journal.pone.0243831)
Supplement: S1 Scheme — (DOCX) [file pone.0243831.s001.docx]

**Scheme 1.** Reagents and conditions: a) (Boc)_2_O, DMAP, *t*-BuOH, rt; b) DCC, DMAP, *t*-BuOH, CH_2_Cl_2_, rt; c) lithium bis(trimethylsilyl)amide, THF, -78°C; d) allyl bromide, THF, -78°C; e) BH_3_, THF, 0°C→rt; f) NaOH, H_2_O_2_, 0°C; g) DAST, DIPEA, CH_2_Cl_2_, -78°C→rt; h) TFA, CH_2_Cl_2_, rt; i) 1-bromo-3-fluoropropane, THF, -78°C.
