# Supplementary material for: Synthesis and analysis of 4-(3-fluoropropyl)-glutamic acid stereoisomers to determine the stereochemical purity of (4S)-4-(3-[18F]fluoropropyl)-L-glutamic acid ([18F]FSPG) for clinical use
Source: PLoS One. 2020 Dec 14;15(12):e0243831. doi: 10.1371/journal.pone.0243831 (PMC7735610; doi:10.1371/journal.pone.0243831)
Supplement: S2 Scheme — (DOCX) [file pone.0243831.s002.docx]

**Scheme 2.** Reagents and conditions: a) lithium bis(trimethylsilyl)amide, THF, -78°C; b) 1-bromo-3-fluoropropane, THF, -78°C→rt; c) allyl bromide, THF, -78°C; d) BH_3_, THF, 0°C; e) NaOH, H_2_O_2_, 0°C; f) DAST, DIPEA, CH_2_Cl_2_, -78°C→rt; g) 3-fluoropropyl triflate, THF, -78°C; h) LiOH, H_2_O, THF, rt; i) HCl, EtOAc, rt; or TFA, CH_2_Cl_2_, rt.
