# Supplementary material for: Synthesis and analysis of 4-(3-fluoropropyl)-glutamic acid stereoisomers to determine the stereochemical purity of (4S)-4-(3-[18F]fluoropropyl)-L-glutamic acid ([18F]FSPG) for clinical use
Source: PLoS One. 2020 Dec 14;15(12):e0243831. doi: 10.1371/journal.pone.0243831 (PMC7735610; doi:10.1371/journal.pone.0243831)
Supplement: S3 Scheme — (DOCX) [file pone.0243831.s003.docx]

**Scheme 3.** Reagents and conditions: a) (4-nitrophenyl)sulfonyl chloride, Et_3_N, CH_2_Cl_2_, rt, 0.5 h; b) [^18^F]KF, K_2_CO_3_, K_2.2.2_, CH_3_CN, 70°C, 5 min; c) 1 N HCl, 120°C, 10 min; d) solid phase extraction: i. H_2_O, ii. saline, iii. Na_2_HPO_4_, NaCl, H_2_O.
