# Supplementary material for: Synthesis and analysis of 4-(3-fluoropropyl)-glutamic acid stereoisomers to determine the stereochemical purity of (4S)-4-(3-[18F]fluoropropyl)-L-glutamic acid ([18F]FSPG) for clinical use
Source: PLoS One. 2020 Dec 14;15(12):e0243831. doi: 10.1371/journal.pone.0243831 (PMC7735610; doi:10.1371/journal.pone.0243831)
Supplement: S1 File — (PDF) [file pone.0243831.s004.pdf]

Supporting Information

Synthesis and analysis of 4-(3-fluoropropyl)-glutamic acid stereoisomers to determine the stereochemical purity of (4*S*)-4-(3-[^{18}F]fluoropropyl)-L-glutamic acid ([^{18}F]FSPG) for clinical use

Kai-Ting Shih,[1¶] Ya-Yao Huang,[2,3,4¶] Chia-Ying Yang,[1] Mei-Fang Cheng,[2] Yu-Wen Tien,[5] Chyng-Yann Shiue,[2,3] Rouh-Fang Yen,[2,3] Ling-Wei Hsin[1,2,3,6*]

[1] School of Pharmacy, College of Medicine, National Taiwan University, Taipei, Taiwan

[2] Department of Nuclear Medicine, National Taiwan University Hospital, Taipei, Taiwan

[3] Molecular Probes Development Core, Molecular Imaging Center, National Taiwan University, Taipei, Taiwan

[4] Institute of Medical Device and Imaging, College of Medicine, National Taiwan University, Taipei Taiwan

[5] Department of Surgery, National Taiwan University Hospital, Taipei, Taiwan

[6] Center for Innovative Therapeutics Discovery, National Taiwan University, Taipei Taiwan.

Table of contents

Page	Contents
S2-S26	^{1}H-, ^{13}C-, and ^{19}F-NMR Spectra.
S27-31	HPLC data for stereoisomers of compounds **3**, **6**, and **1**.
S32-35	Radiosynthesis of [^{18}F]FSPG

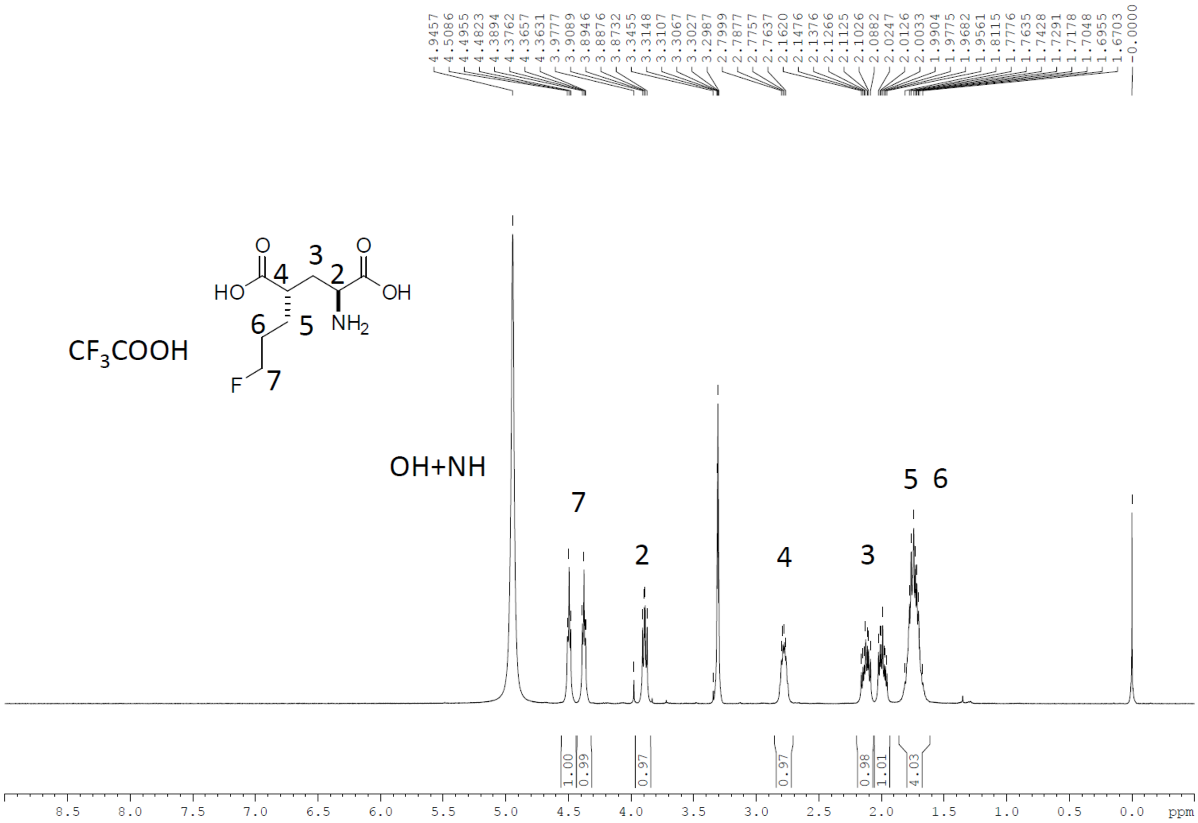

Figure 1. ^{1}H-NMR spectrum of compound (2*S*,4*S*)-**1** (FSPG, 400 MHz, methanol-*d*$_4$).

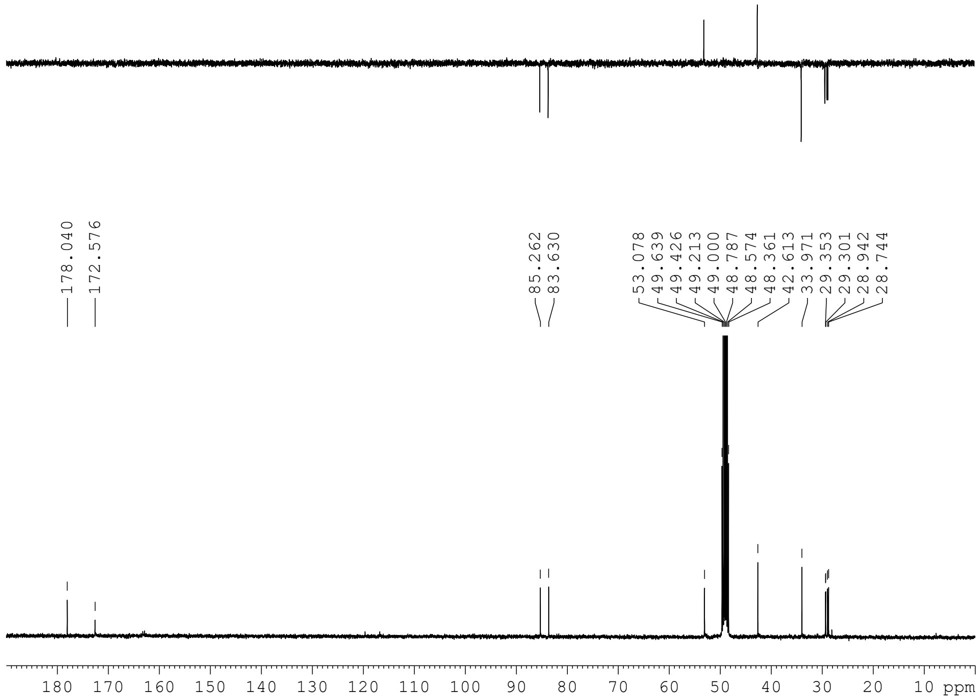

Figure 2. ^{13}C-NMR spectra of compound (2*S*,4*S*)-**1** (FSPG, 100 MHz, methanol-*d*$_4$).

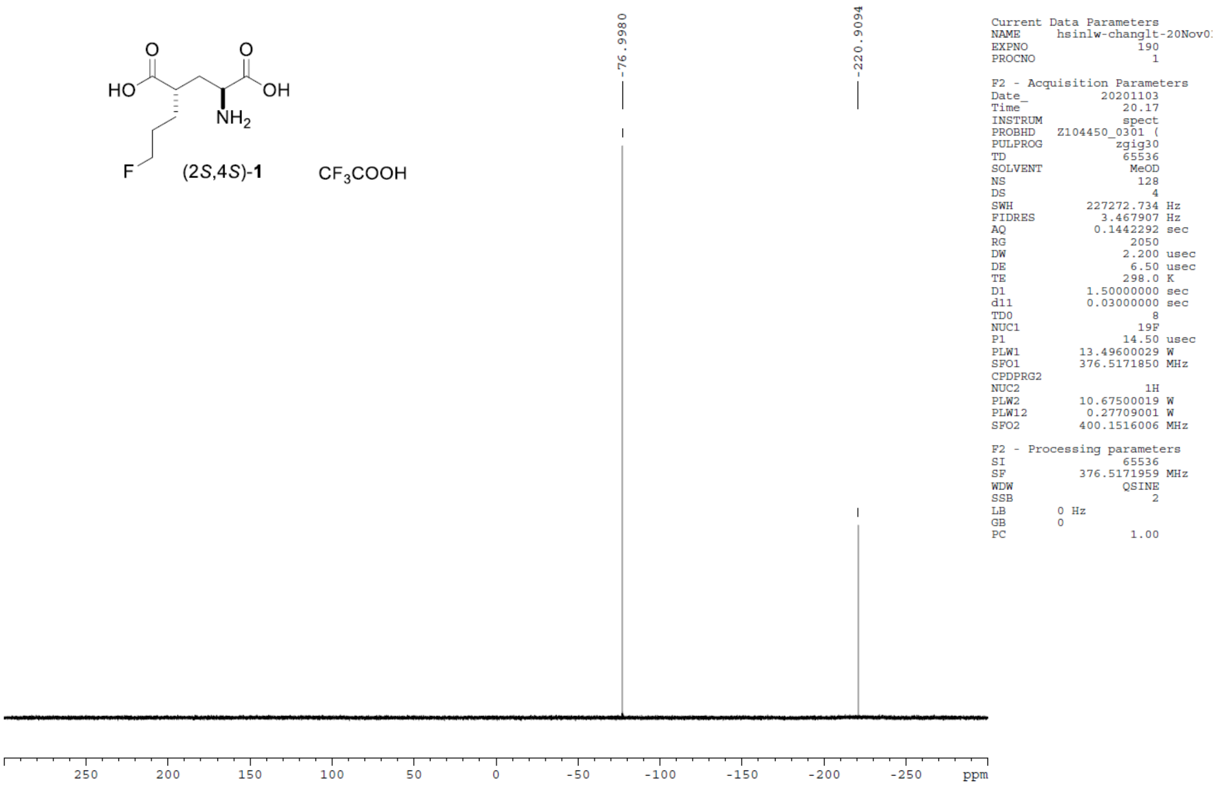

Figure 3. ^{19}F-NMR spectrum of compound (2*S*,4*S*)-**1** (FSPG, 377 MHz, methanol-d_4).

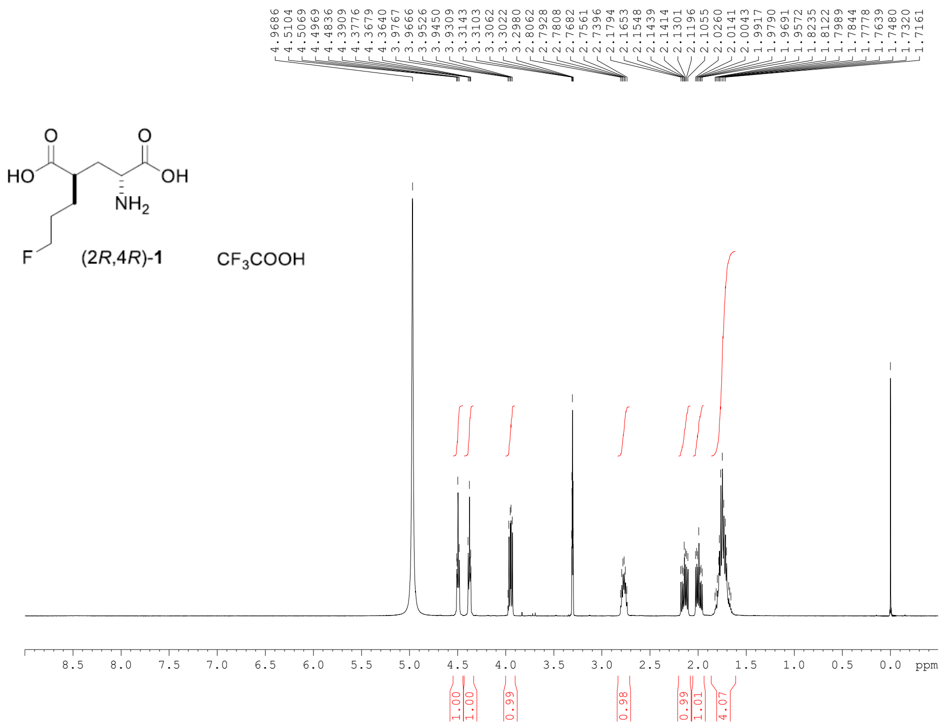

Figure 4. ¹H-NMR spectrum of compound (2*R*,4*R*)-**1** (400 MHz, methanol-*d*4).

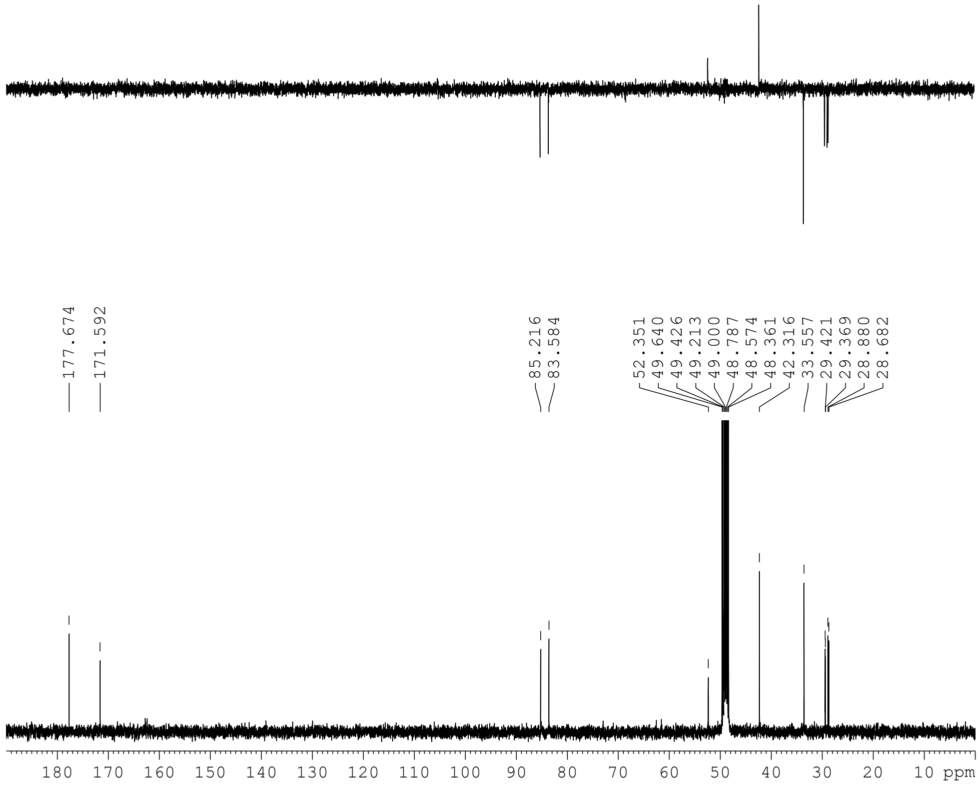

Figure 5. ¹³C-NMR spectra of compound (2*R*,4*R*)-**1** (100 MHz, methanol-*d*4).

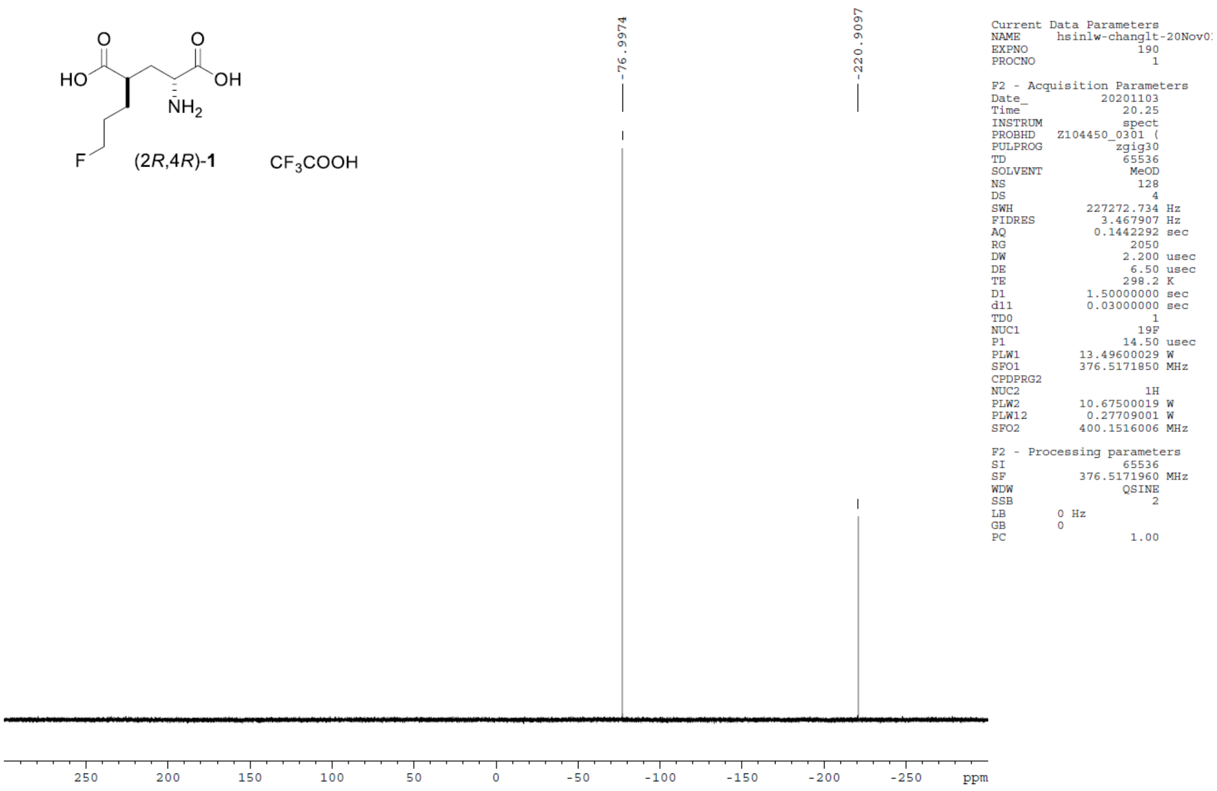

Figure 6. ^{19}F-NMR spectrum of compound (2*R*,4*R*)-**1** (377 MHz, methanol-d_4).

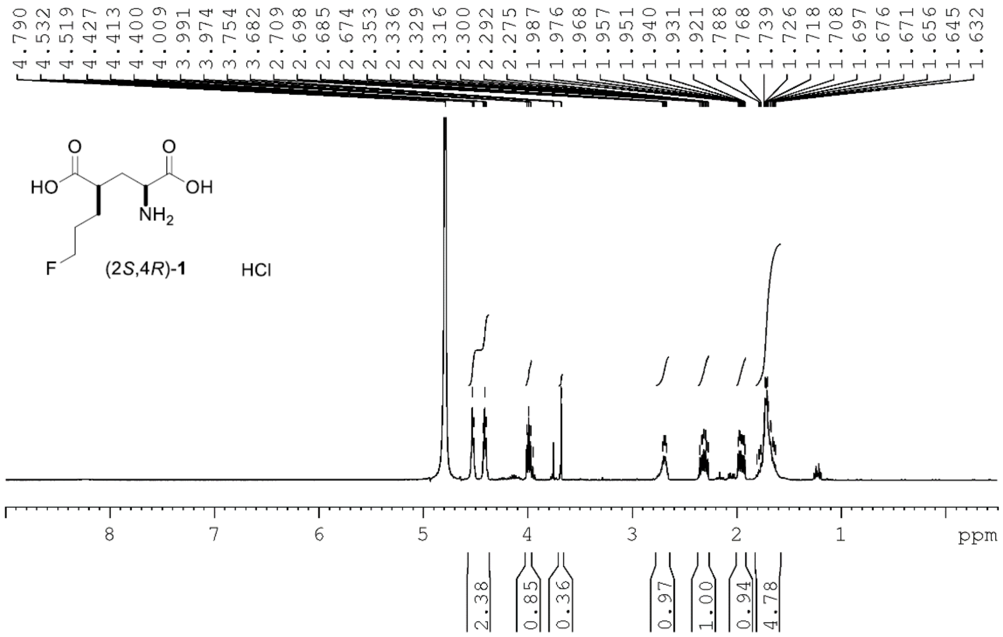

Figure 7. ^{1}H-NMR spectrum of compound (2*S*,4*R*)-**1** (400 MHz, D_2O).

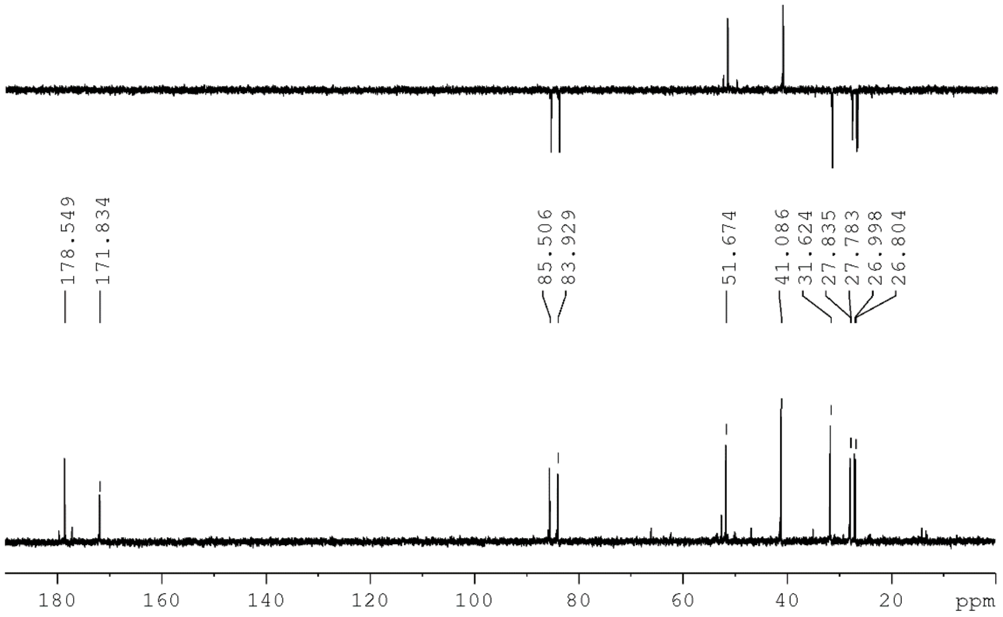

Figure 8. ^{13}C-NMR spectra of compound (2*S*,4*R*)-**1** (100 MHz, D_2O).

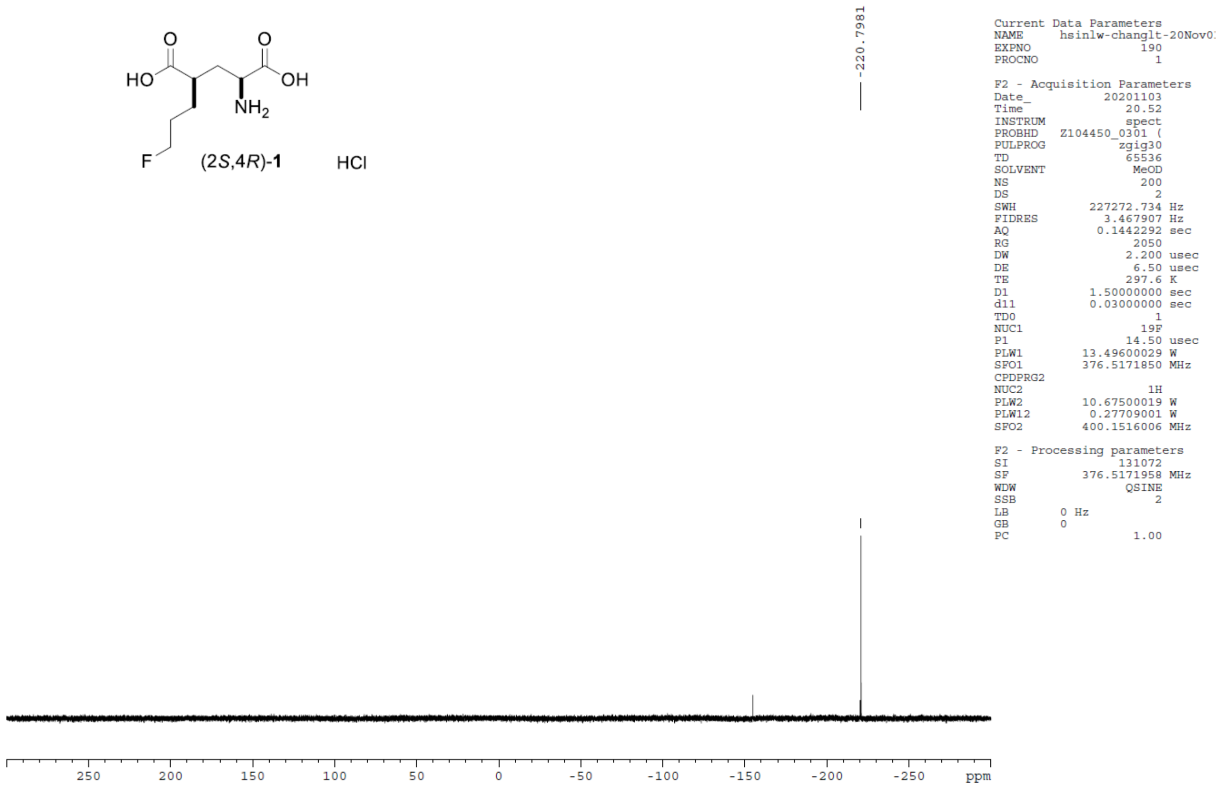

Figure 9. ^{19}F-NMR spectrum of compound (2*S*,4*R*)-**1** (377 MHz, methanol-d_4).

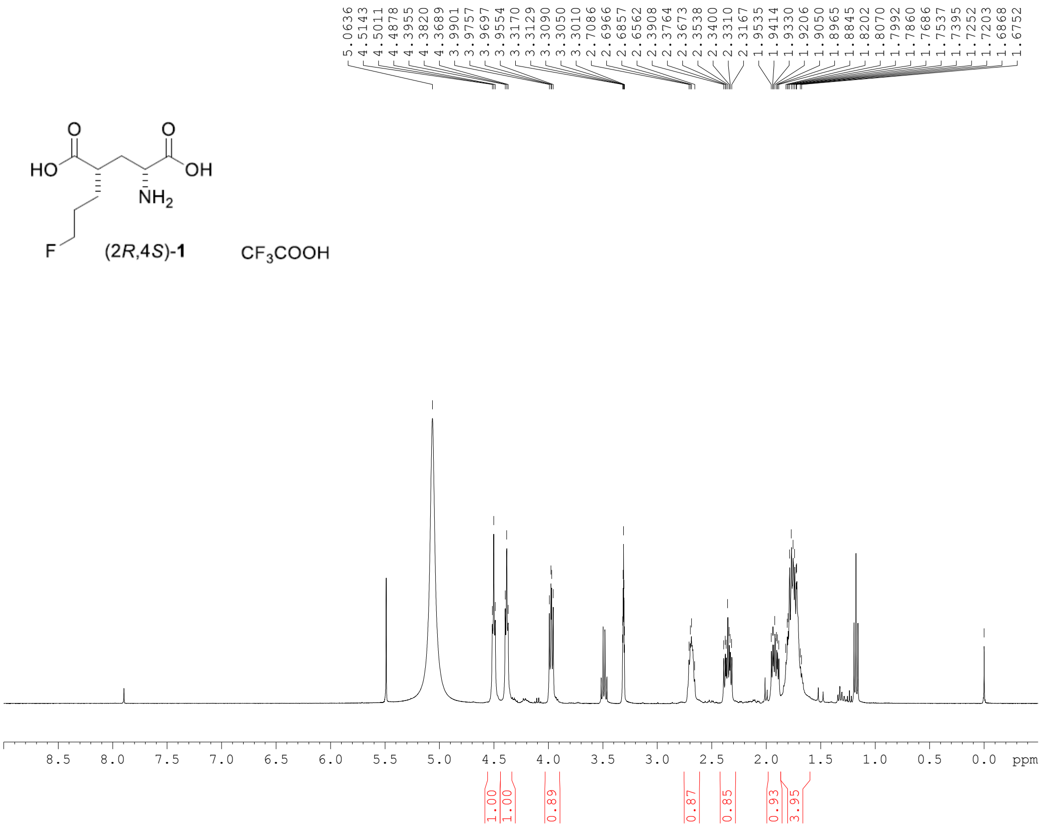

Figure 10. ^{1}H-NMR spectrum of compound (2*R*,4*S*)-**1** (400 MHz, methanol-*d*4).

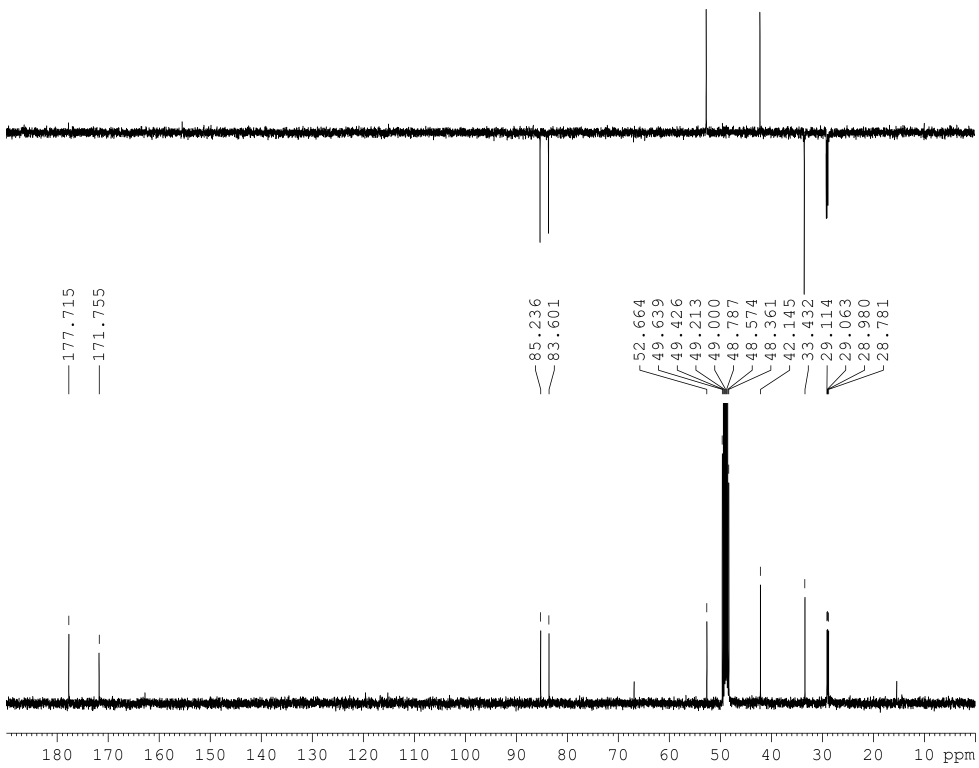

Figure 11. ^{13}C-NMR spectra of compound (2*R*,4*S*)-**1** (100 MHz, methanol-*d*4).

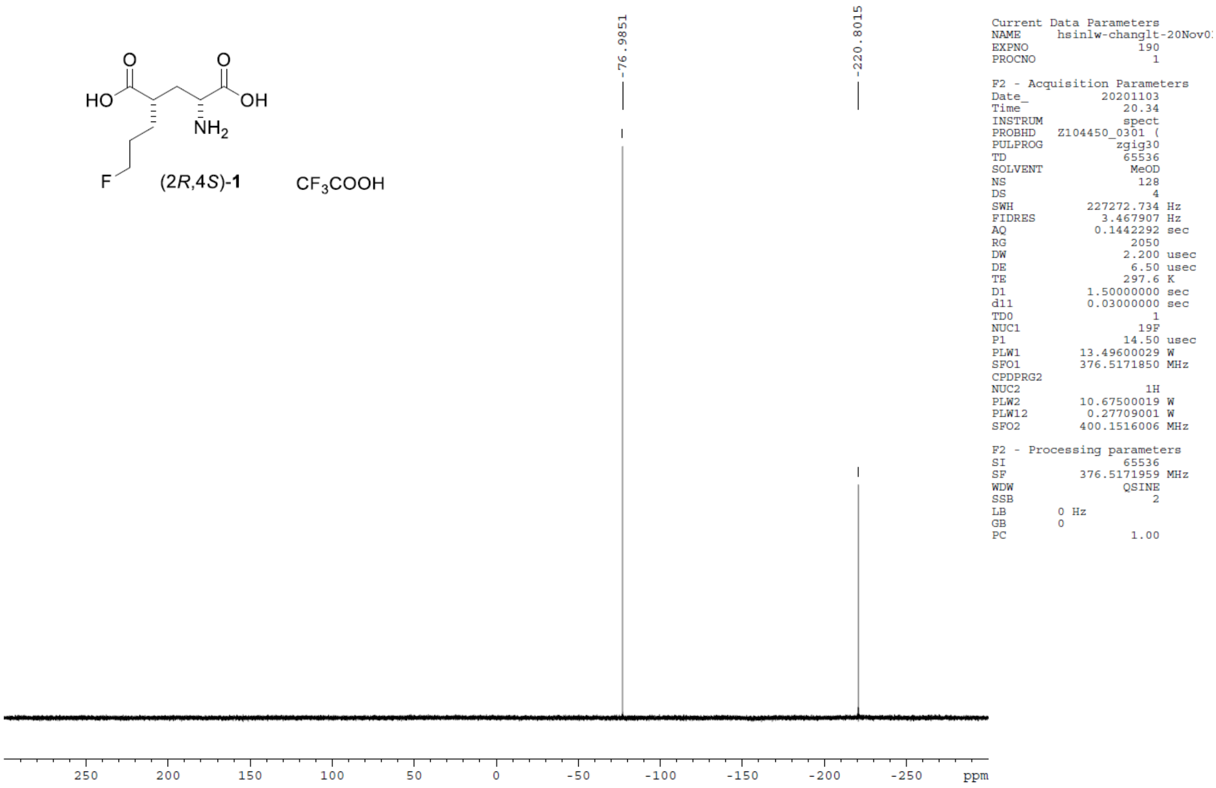

Figure 12. ^{19}F-NMR spectrum of compound (2*R*,4*S*)-**1** (377 MHz, methanol-d_4).

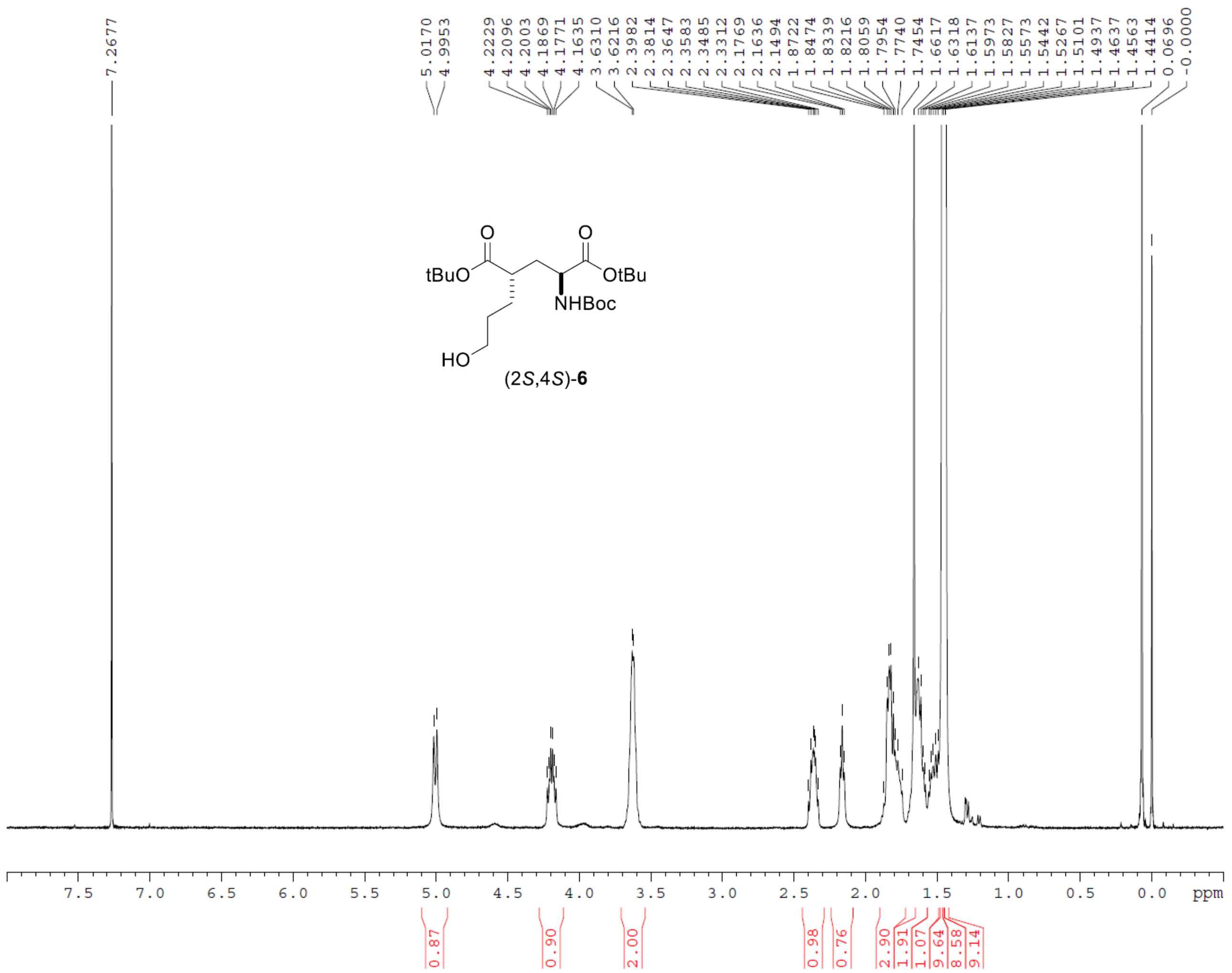

Figure 13. ^{1}H-NMR spectrum of compound (2*S*,4*S*)-**6** (400 MHz, $CDCl_3$).

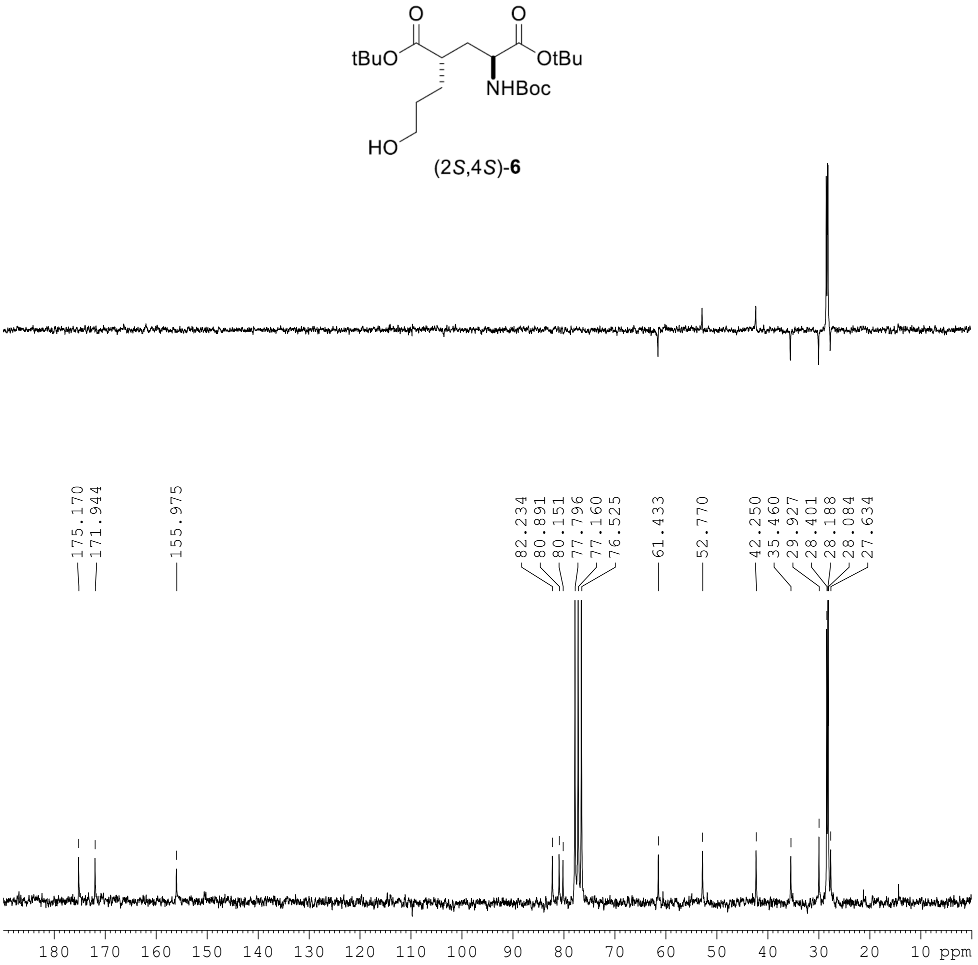

Figure 14. ^{13}C-NMR spectra of compound (2*S*,4*S*)-**6** (50 MHz, $CDCl_3$).

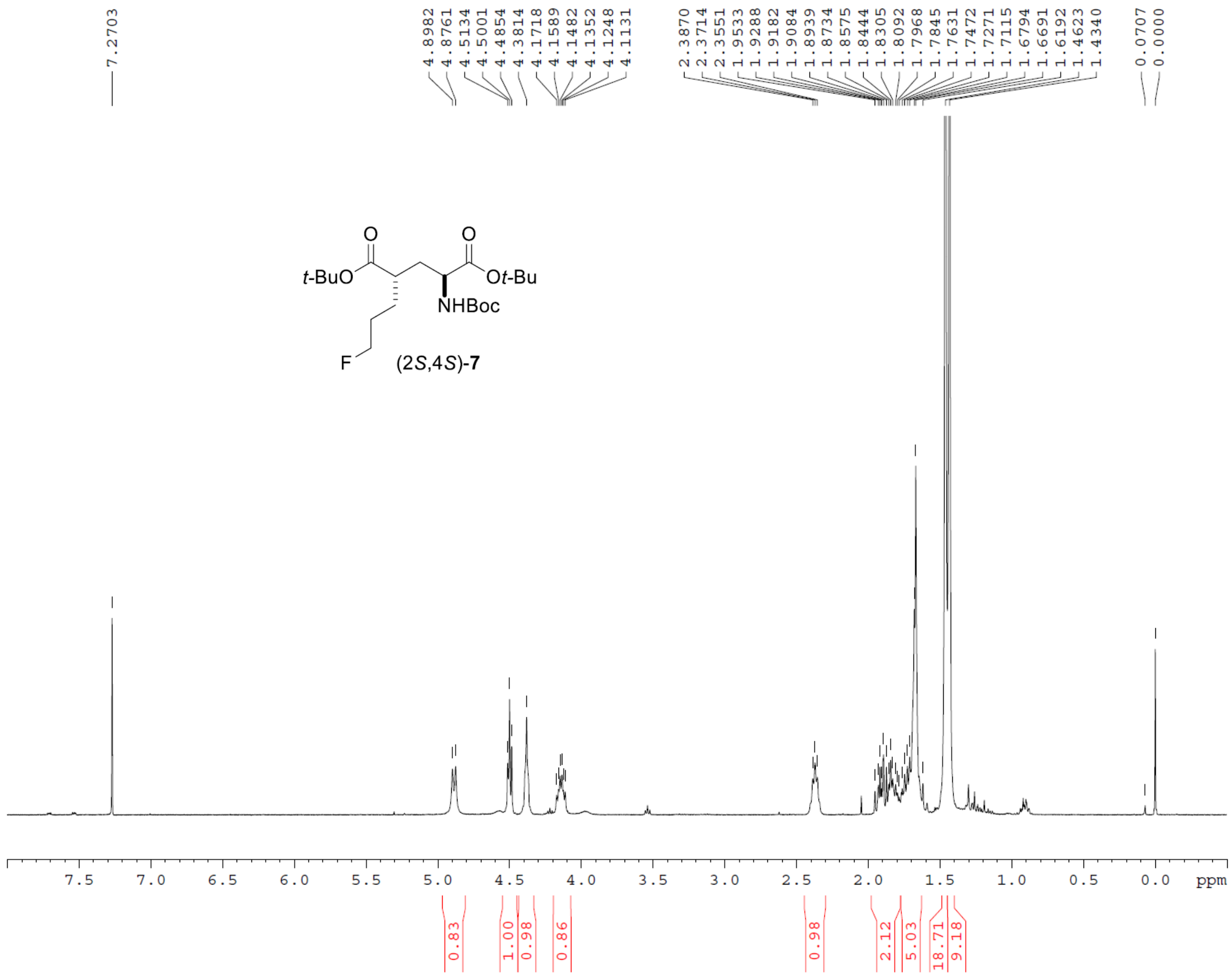

Figure 15. ^{1}H-NMR spectrum of compound (2*S*,4*S*)-**7** (400 MHz, $CDCl_3$).

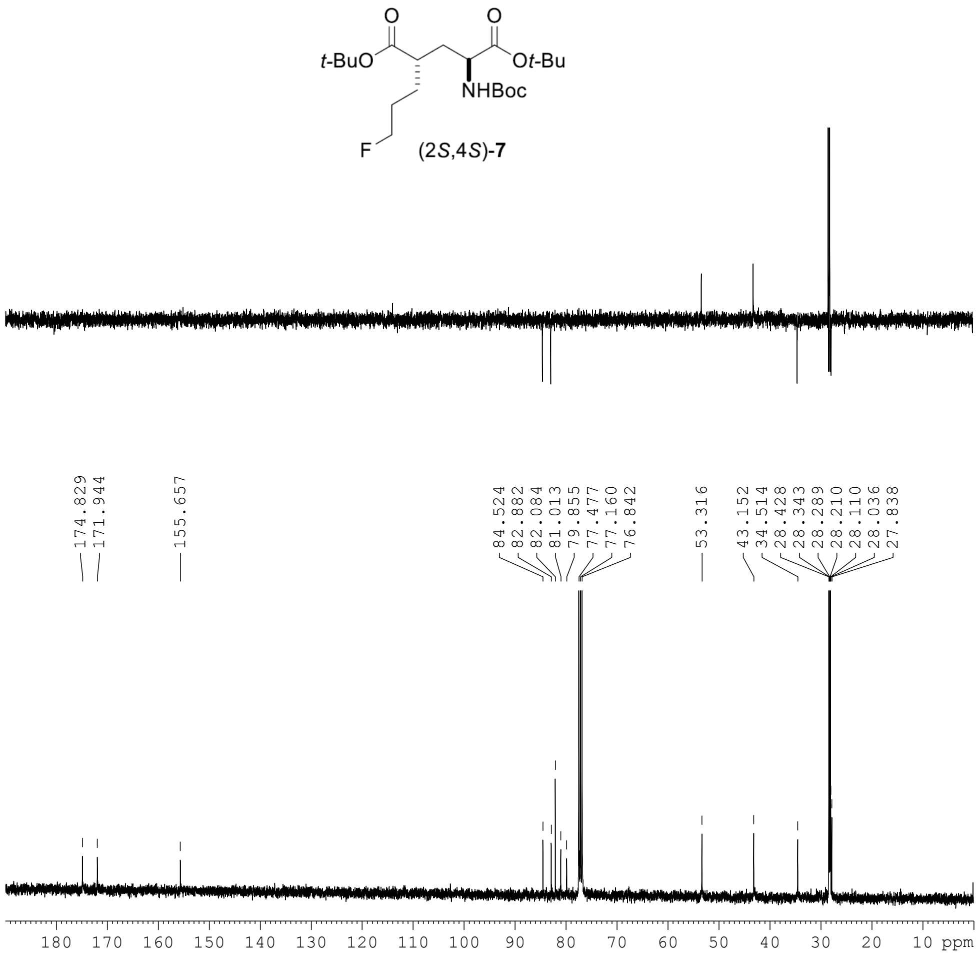

Figure 16. ^{13}C-NMR spectra of compound (2*S*,4*S*)-**7** (100 MHz, $CDCl_3$).

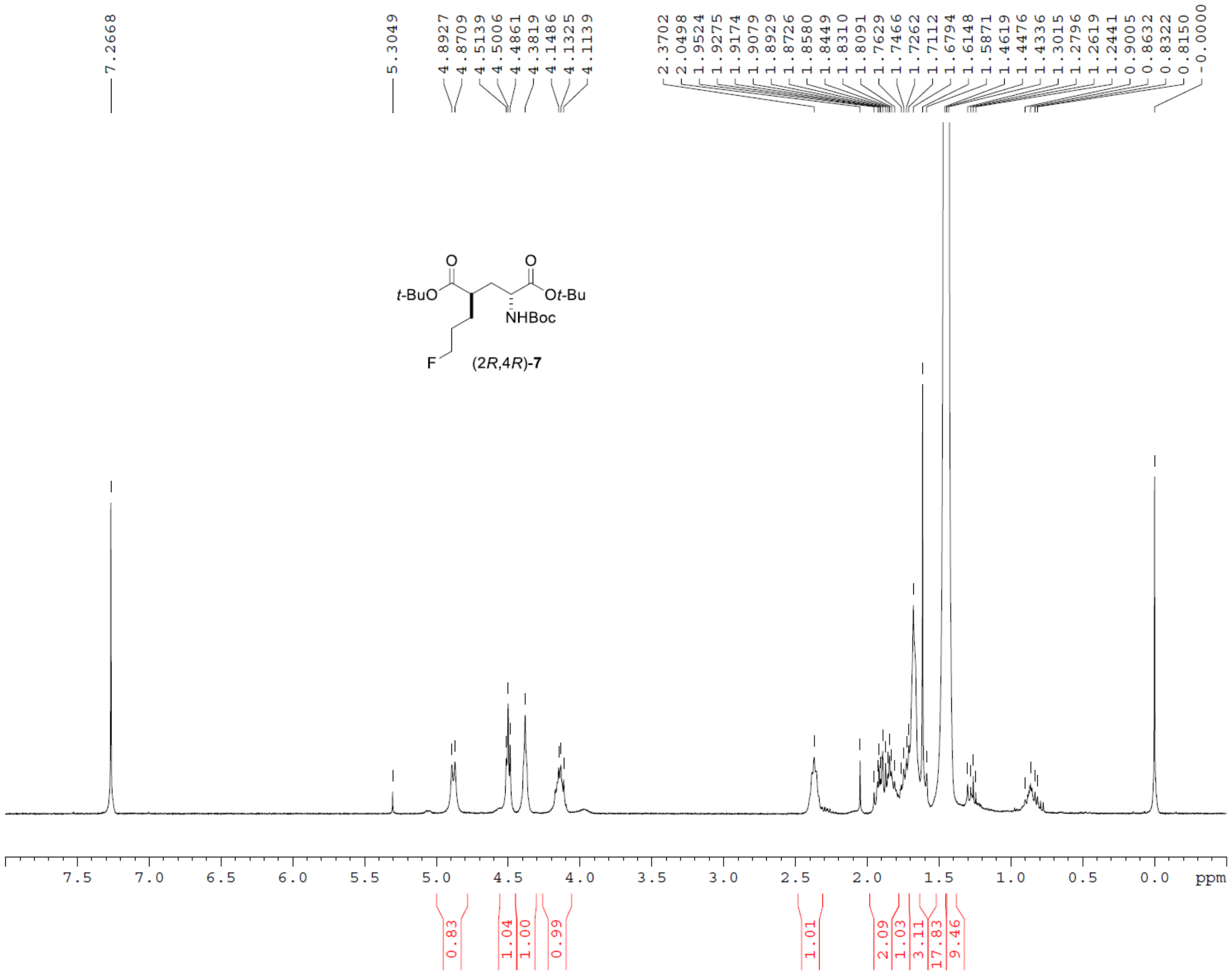

Figure 17. ^{1}H-NMR spectrum of compound (2*R*,4*R*)-**7** (400 MHz, $CDCl_3$).

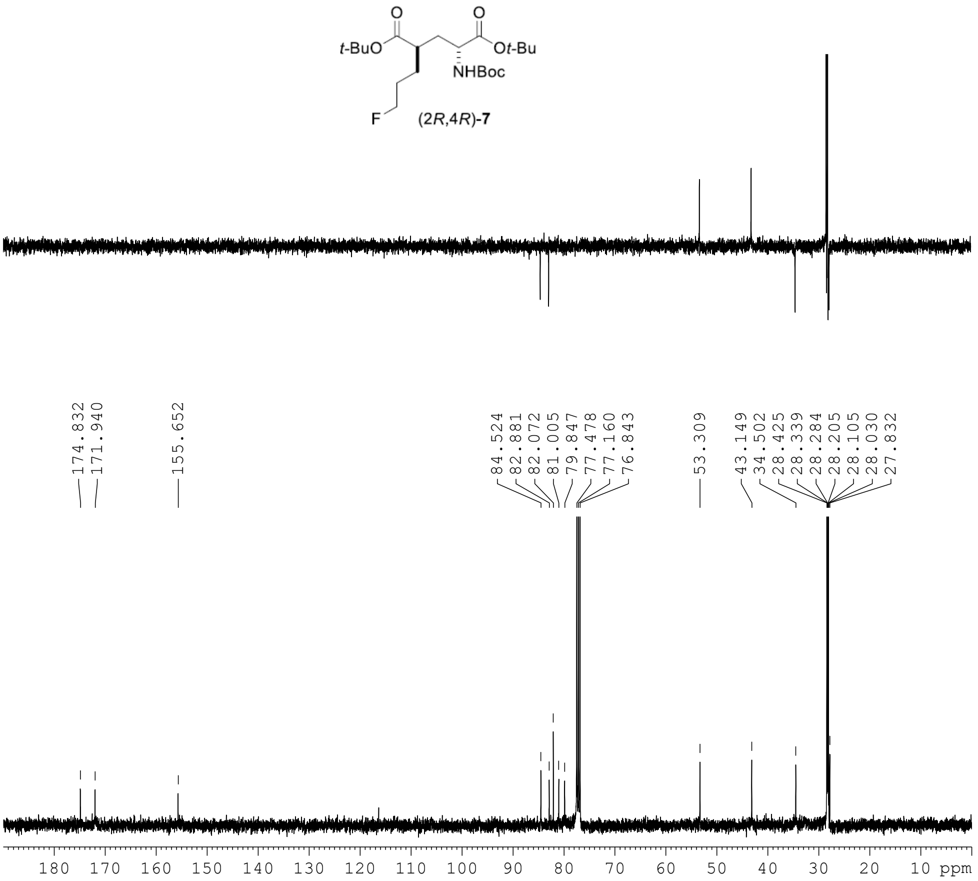

Figure 18. ^{13}C-NMR spectra of compound (2*R*,4*R*)-**7** (100 MHz, $CDCl_3$).

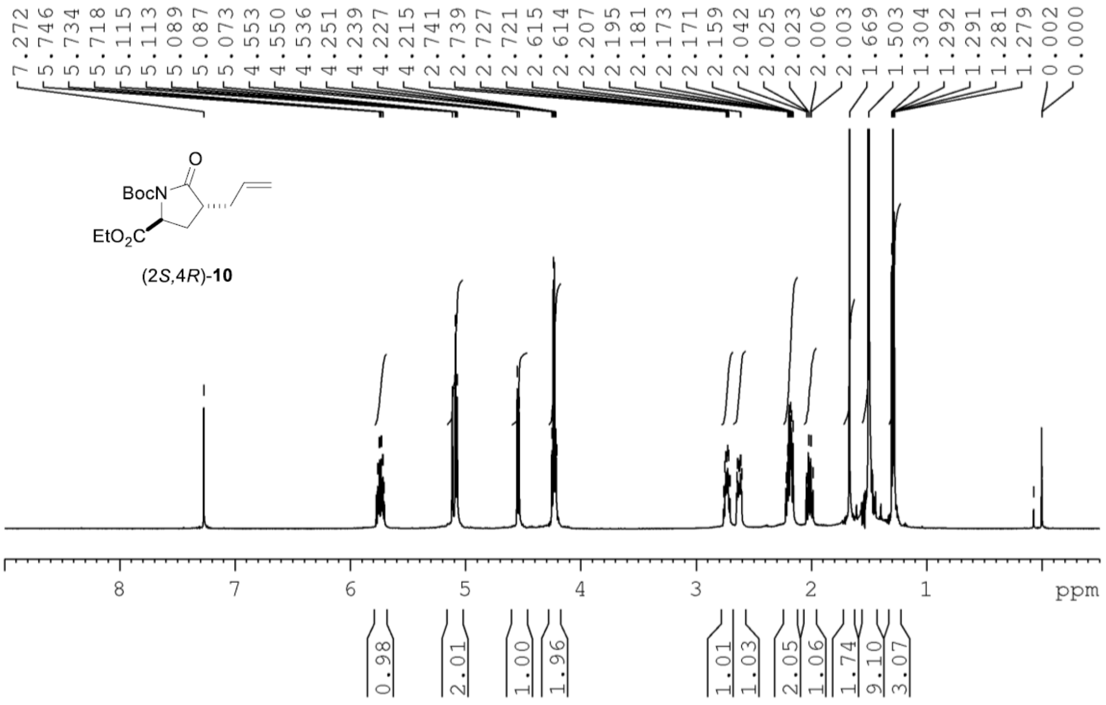

Figure 19. ^{1}H-NMR spectrum of compound (2*S*,4*R*)-**10** (600 MHz, $CDCl_3$).

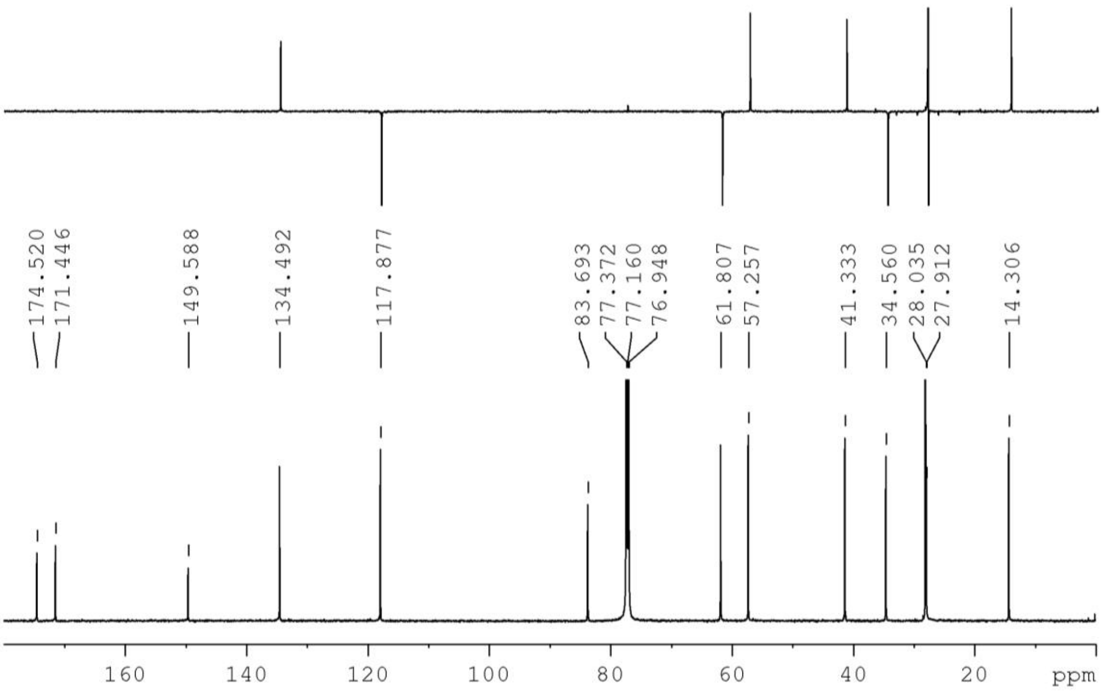

Figure 20. ^{13}C-NMR spectra of compound (2*S*,4*R*)-**10** (150 MHz, $CDCl_3$).

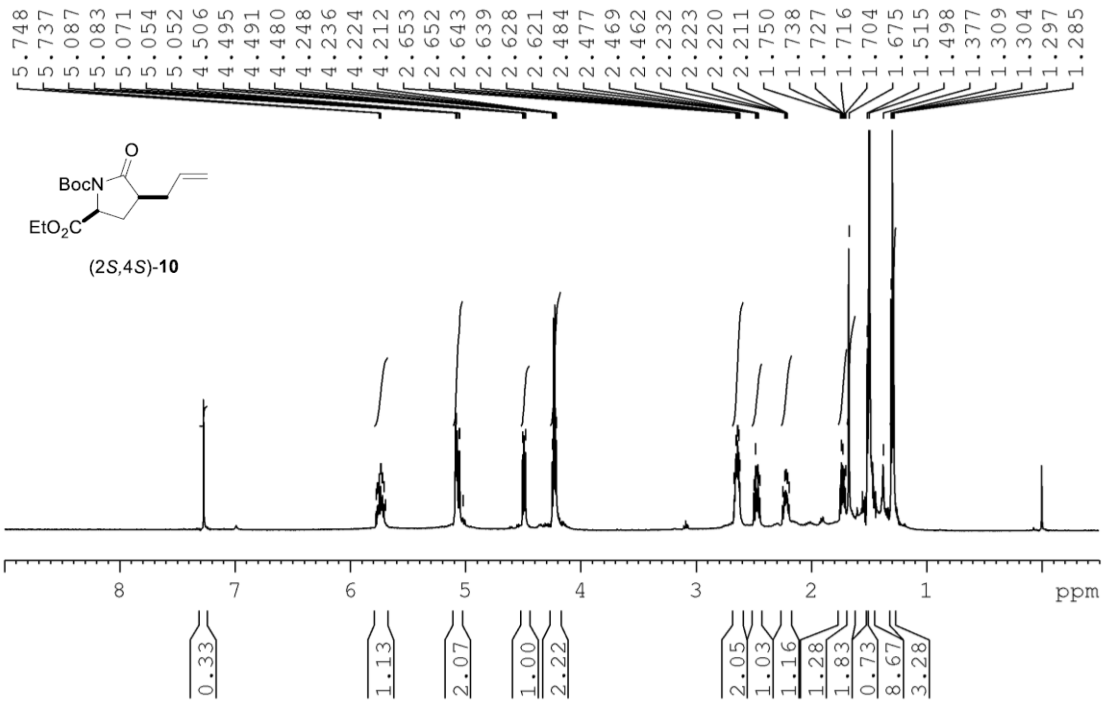

Figure 21. ^{1}H-NMR spectrum of compound (2*S*,4*S*)-**10** (600 MHz, $CDCl_3$).

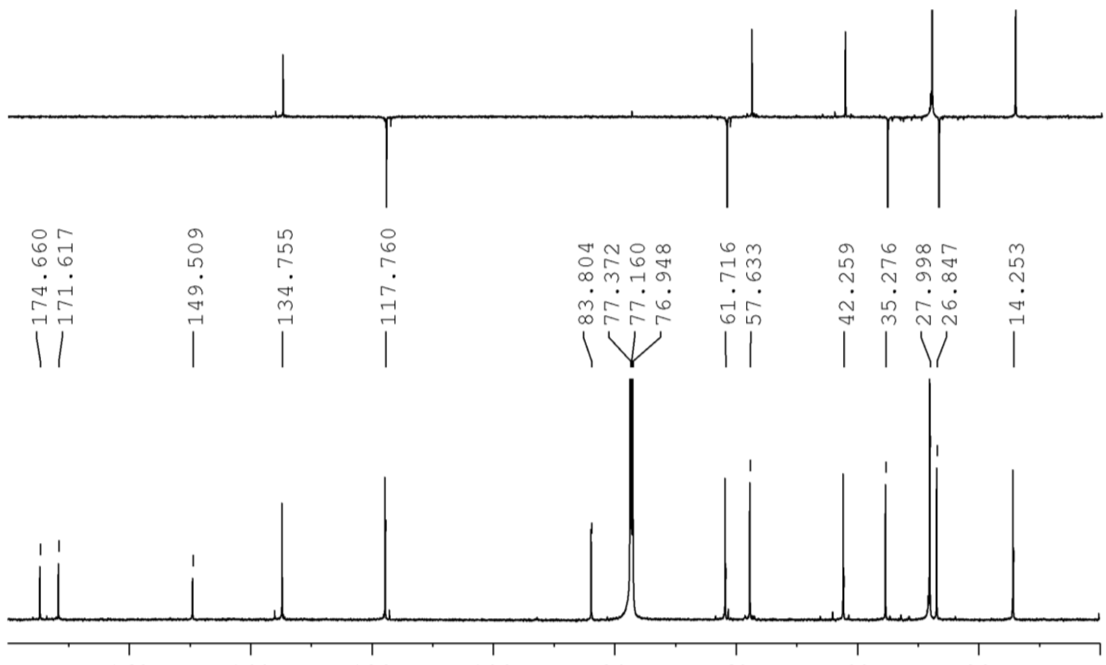

Figure 22. ^{13}C-NMR spectra of compound (2*S*,4*S*)-**10** (150 MHz, $CDCl_3$).

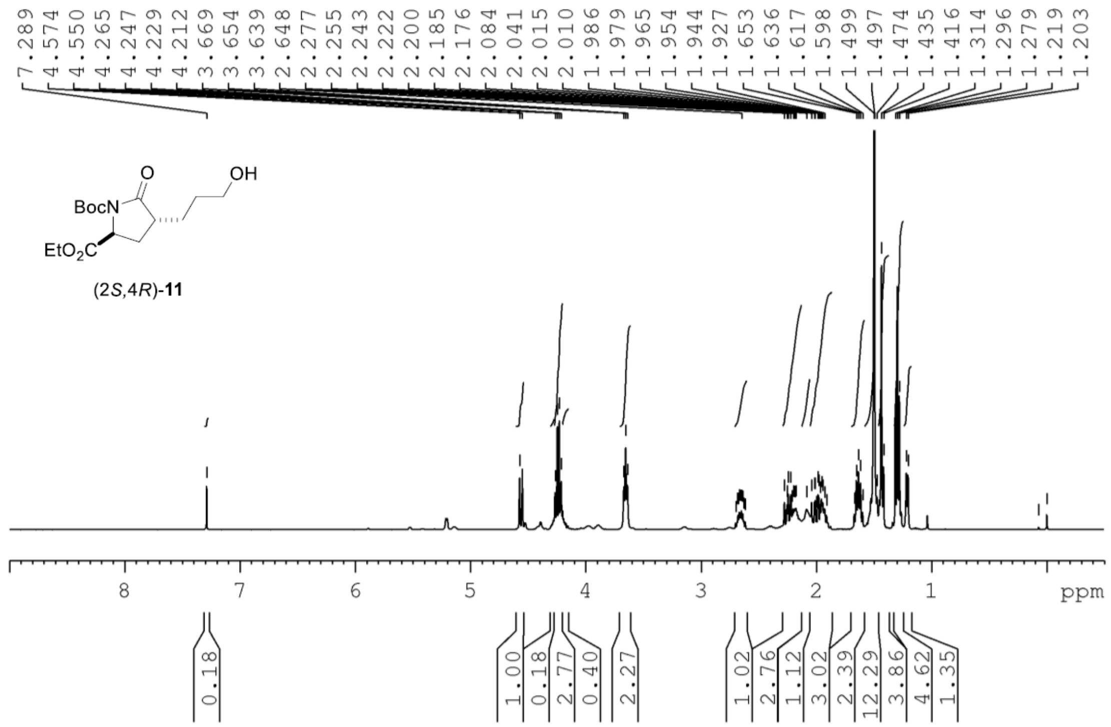

Figure 23. ^{1}H-NMR spectrum of compound (2*S*,4*R*)-**11** (400 MHz, $CDCl_3$).

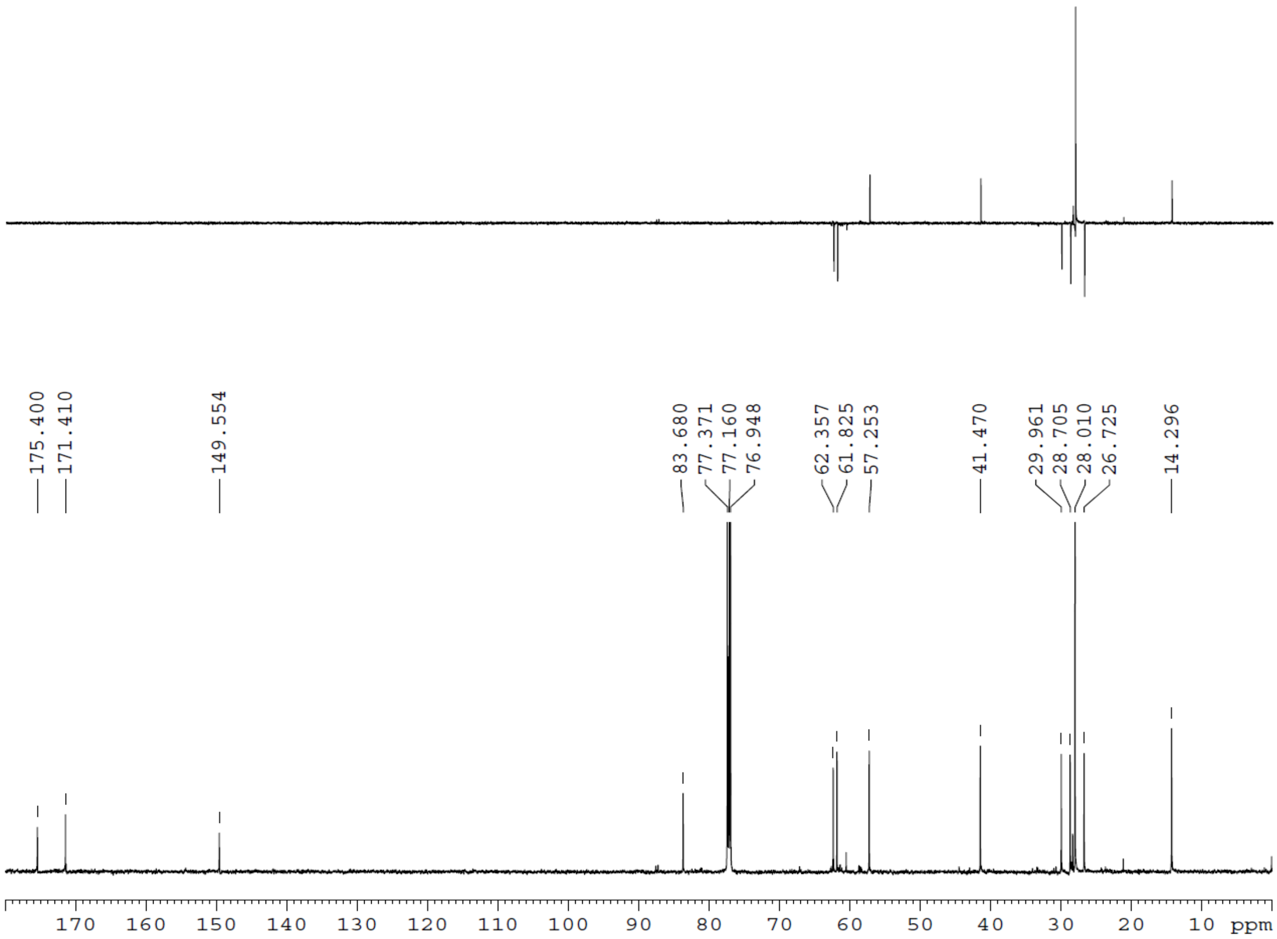

Figure 24. ^{13}C-NMR spectra of compound (2*S*,4*R*)-**11** (150 MHz, $CDCl_3$).

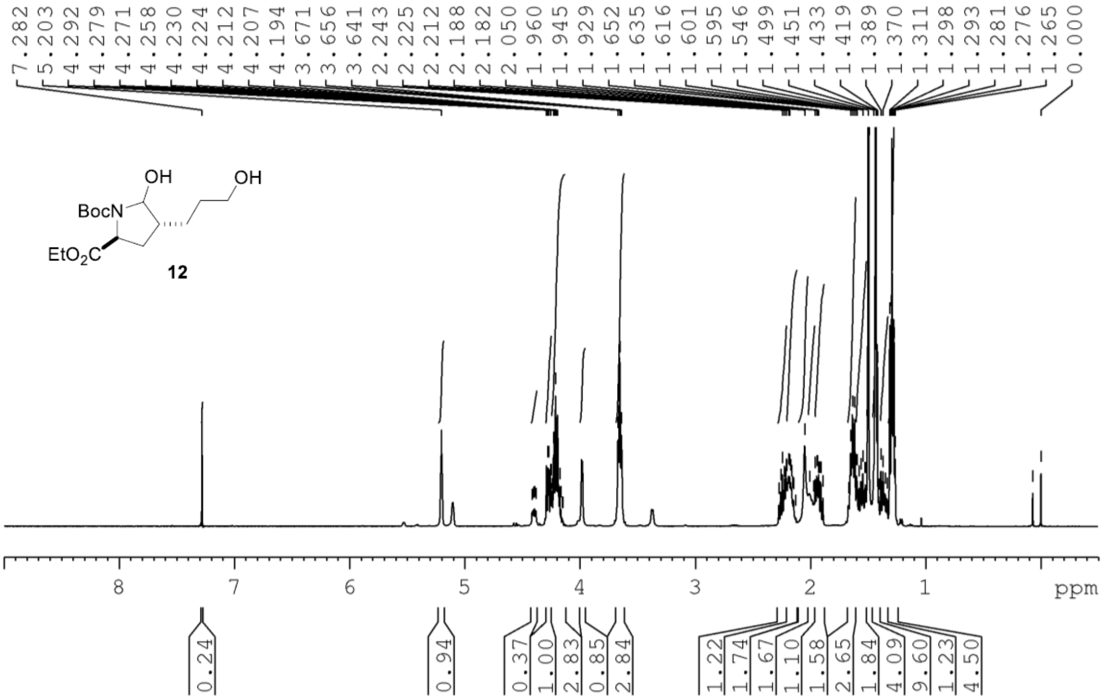

Figure 25. ^{1}H-NMR spectrum of compound **12** (400 MHz, $CDCl_3$).

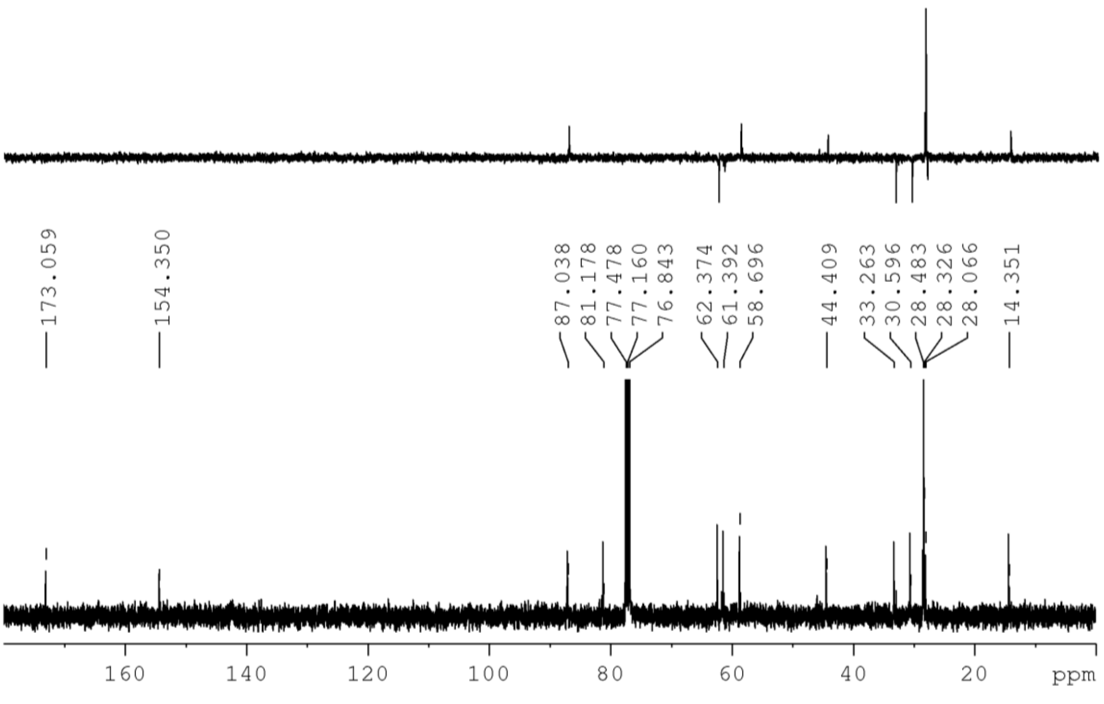

Figure 26. ^{13}C-NMR spectra of compound **12** (100 MHz, $CDCl_3$).

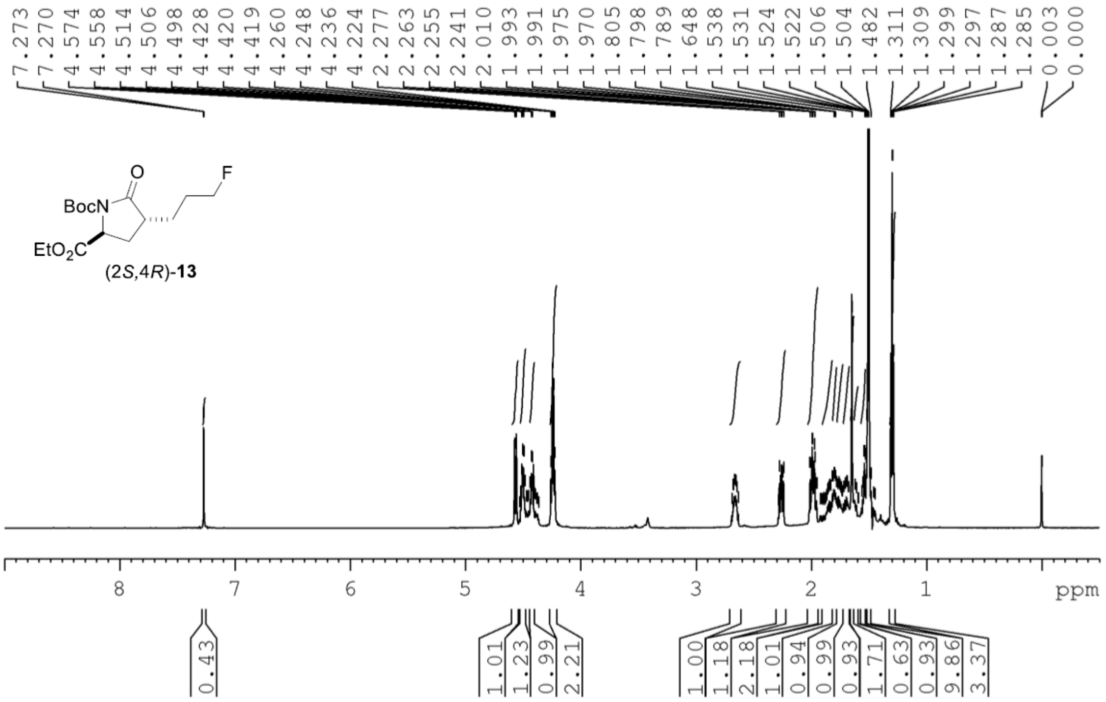

Figure 27. ^{1}H-NMR spectrum of compound (2*S*,4*R*)-**13** (600 MHz, $CDCl_3$).

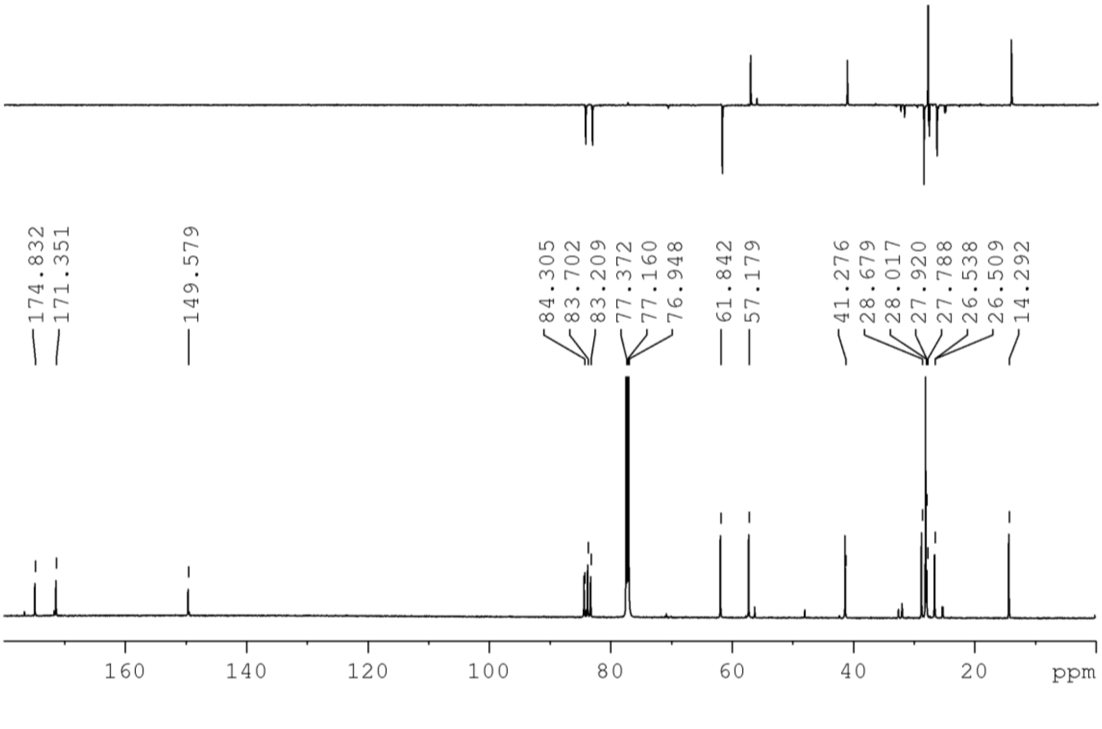

Figure 28. ^{13}C-NMR spectra of compound (2*S*,4*R*)-**13** (150 MHz, $CDCl_3$).

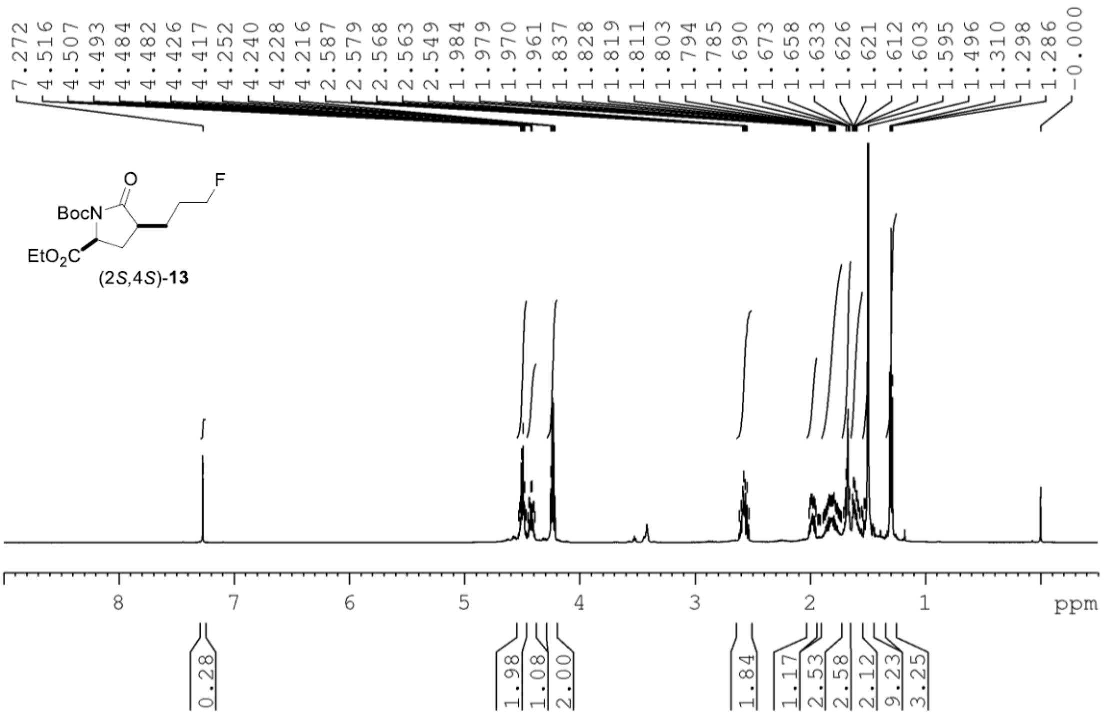

Figure 29. ^{1}H-NMR spectrum of compound (2*S*,4*S*)-**13** (600 MHz, $CDCl_3$).

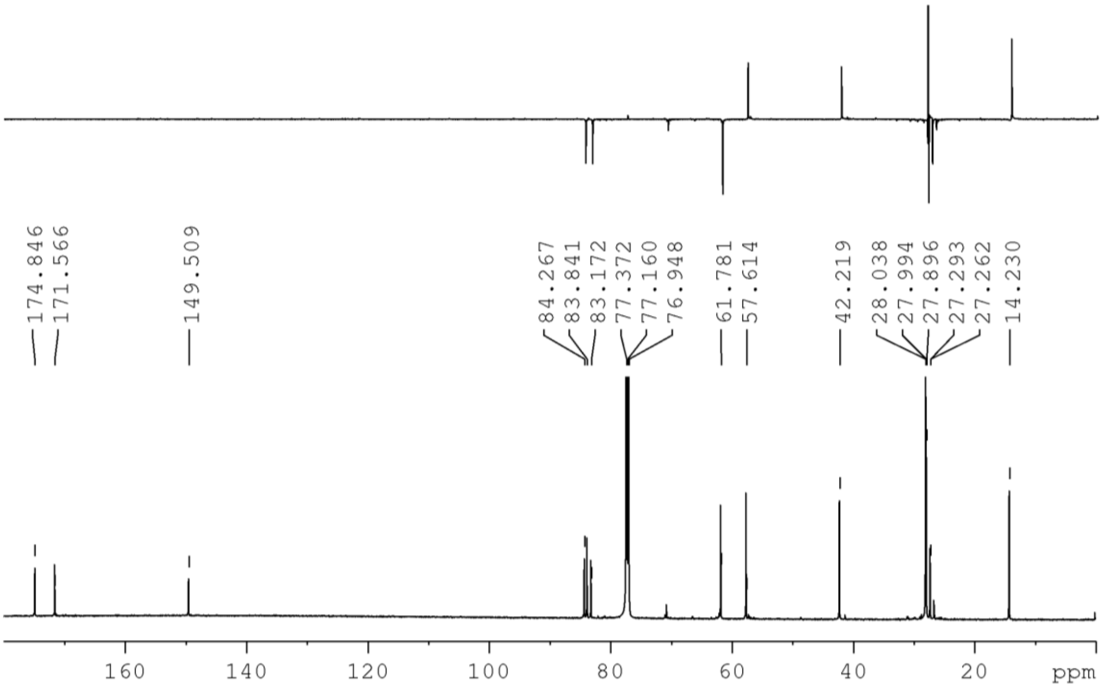

Figure 30. ^{13}C-NMR spectra of compound (2*S*,4*S*)-**13** (100 MHz, $CDCl_3$).

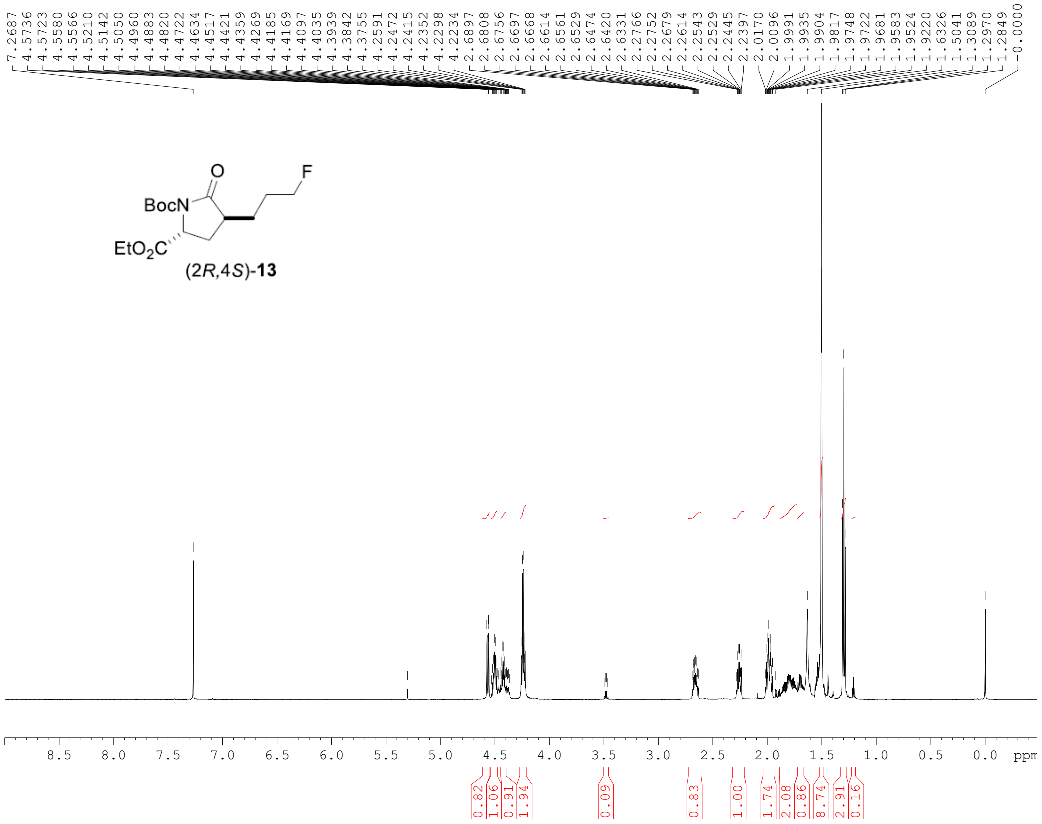

Figure 31. ^{1}H-NMR spectrum of compound (2*R*,4*S*)-**13** (600 MHz, $CDCl_3$).

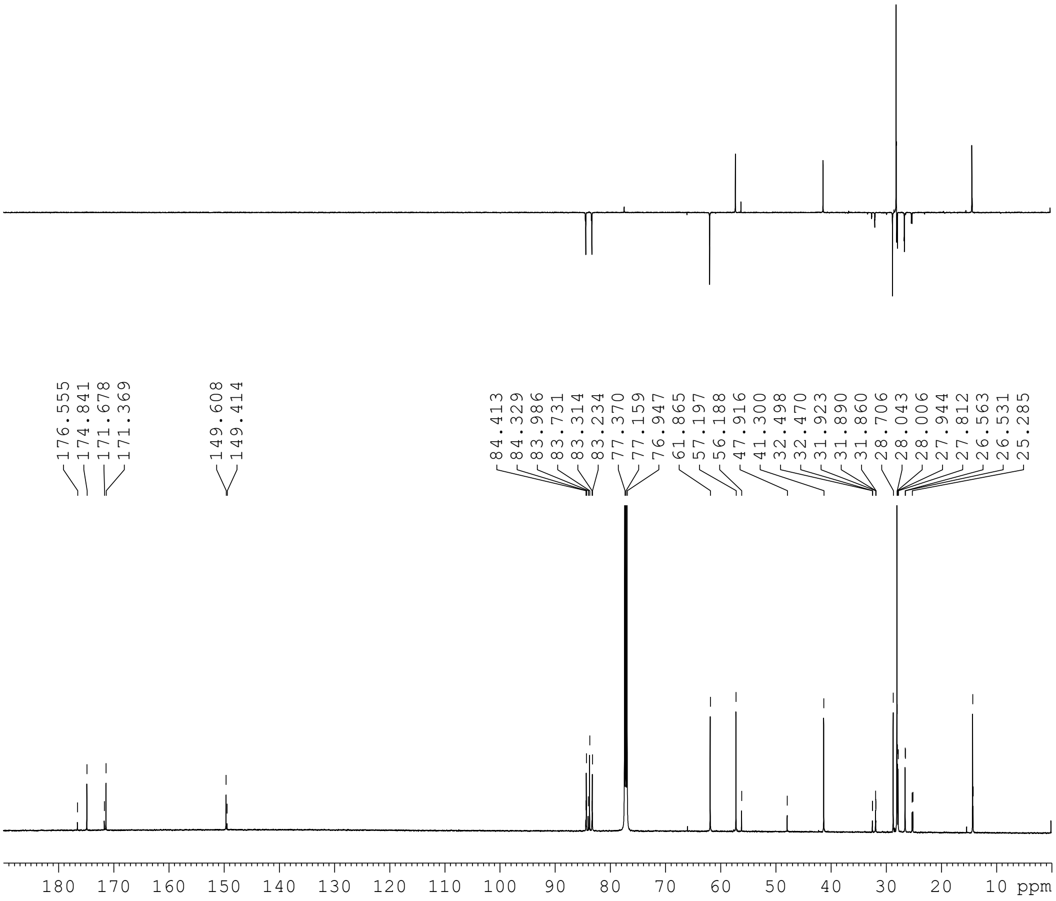

Figure 32. ^{13}C-NMR spectra of compound (2*R*,4*S*)-**13** (150 MHz, $CDCl_3$).

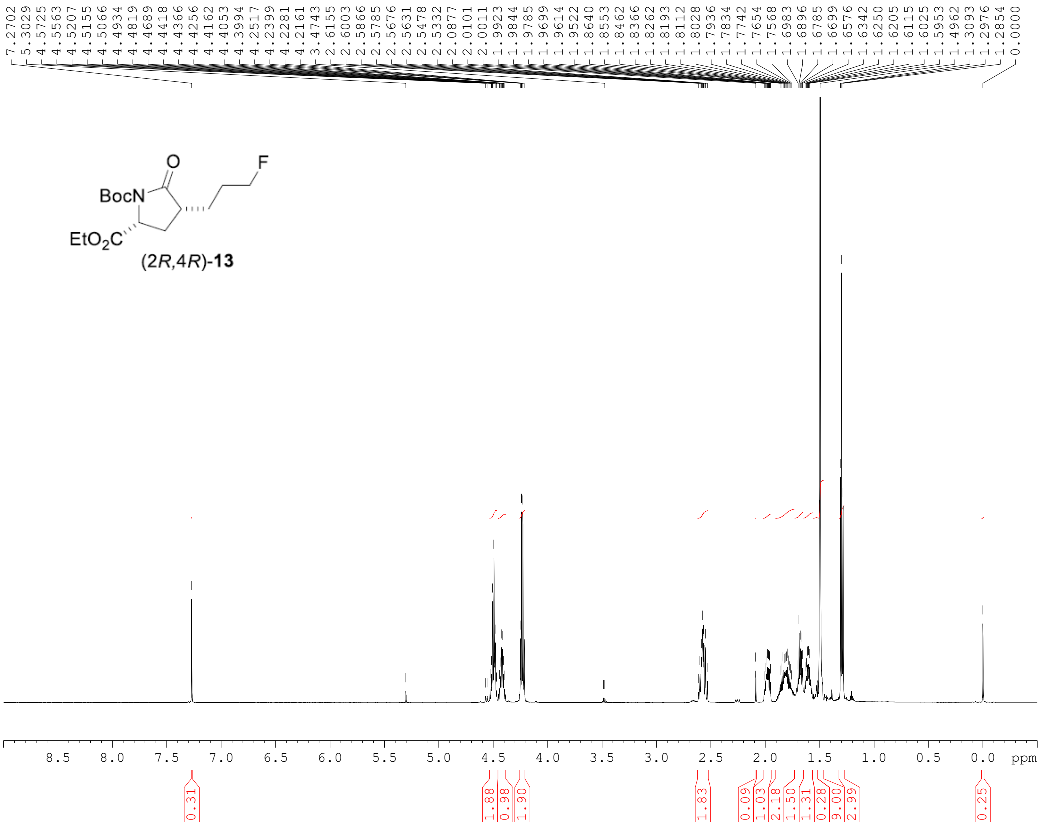

Figure 33. ^{1}H-NMR spectrum of compound (2*R*,4*R*)-**13** (400 MHz, $CDCl_3$).

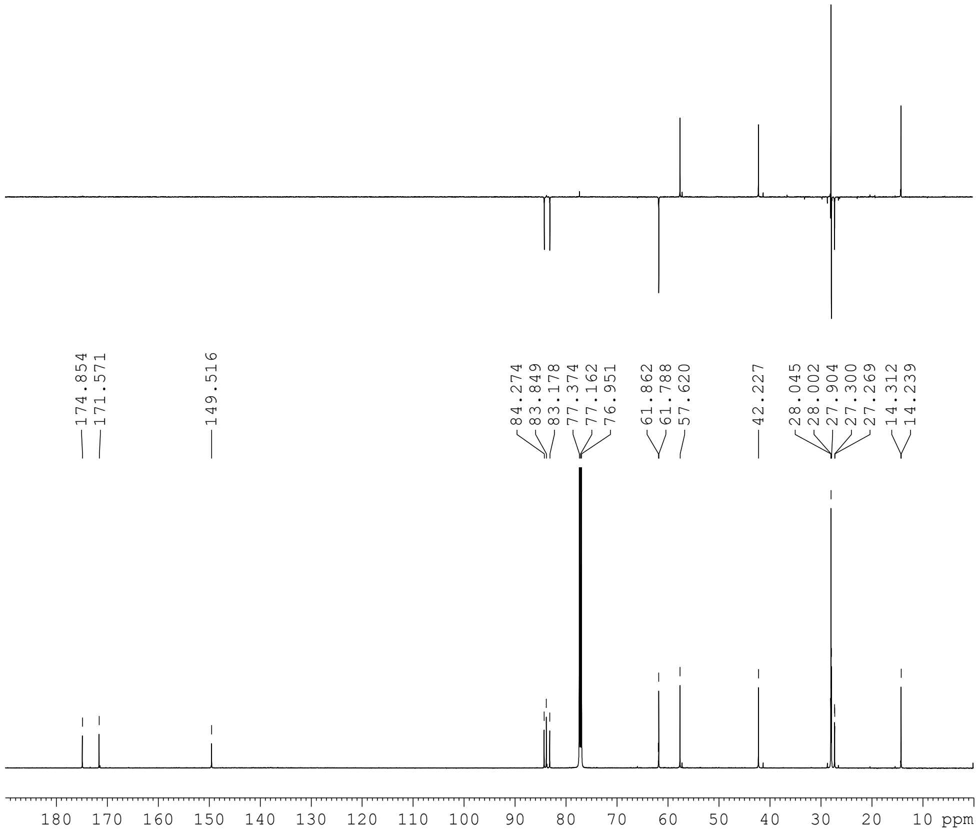

Figure 34. ^{13}C-NMR spectra of compound (2*R*,4*R*)-**13** (100 MHz, $CDCl_3$).

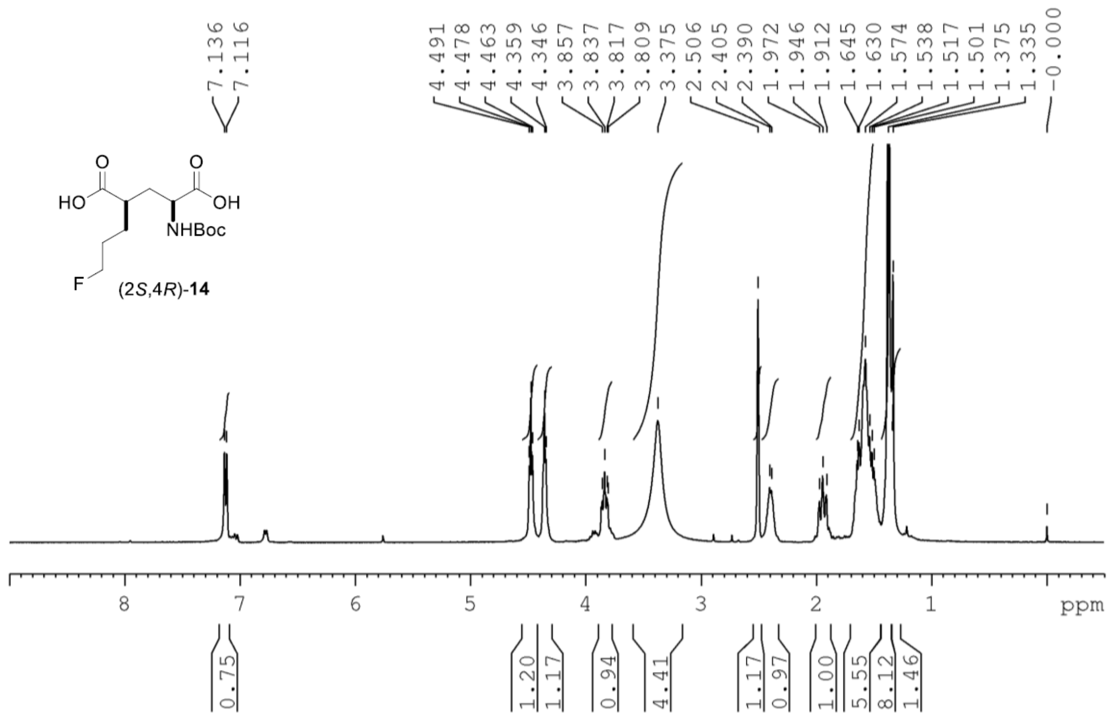

Figure 35. ^{1}H-NMR spectrum of compound (2*S*,4*R*)-**14** (400 MHz, DMSO-d_6).

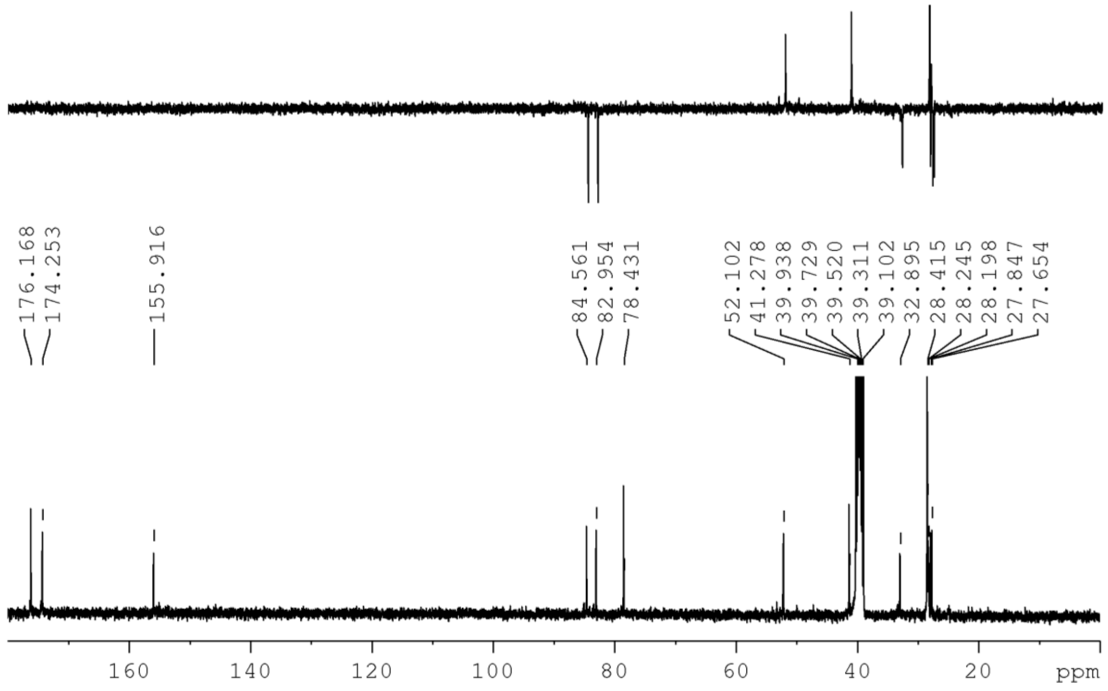

Figure 36. ^{13}C-NMR spectra of compound (2*S*,4*R*)-**14** (100 MHz, DMSO-d_6).

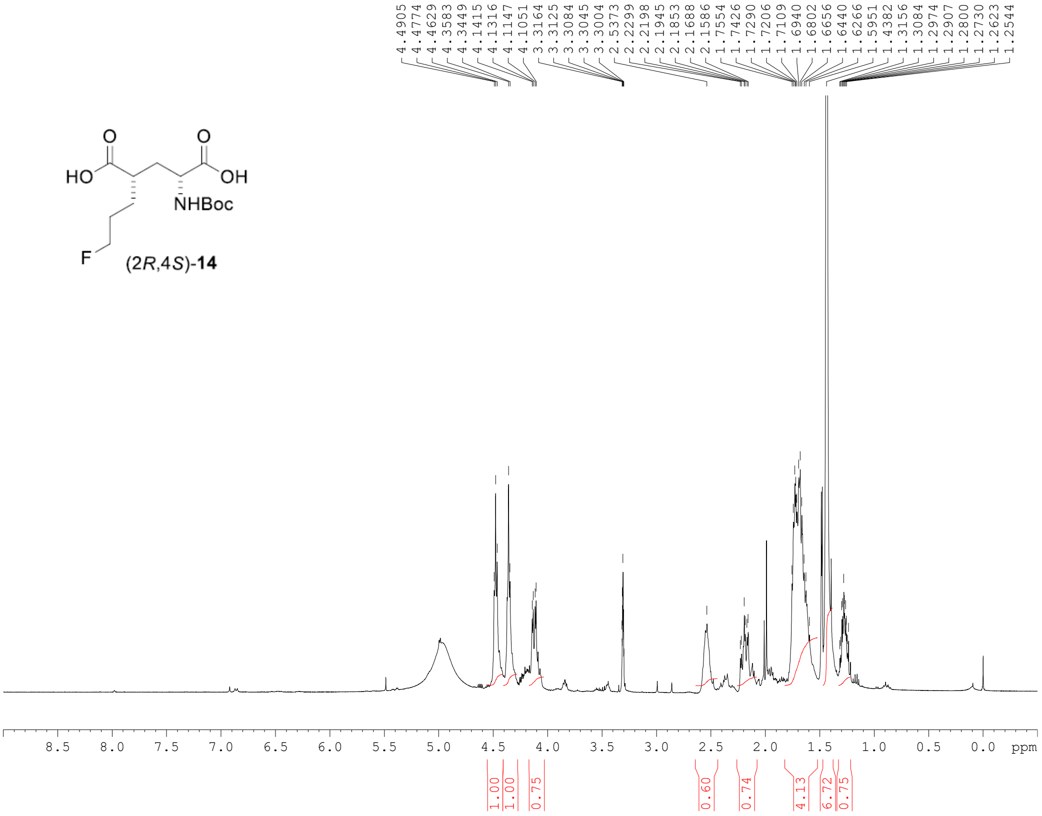

(2*R*,4*S*)-**14**

Figure 37. ^{1}H-NMR spectrum of compound (2*R*,4*S*)-**14** (400 MHz, methanol-d_4).

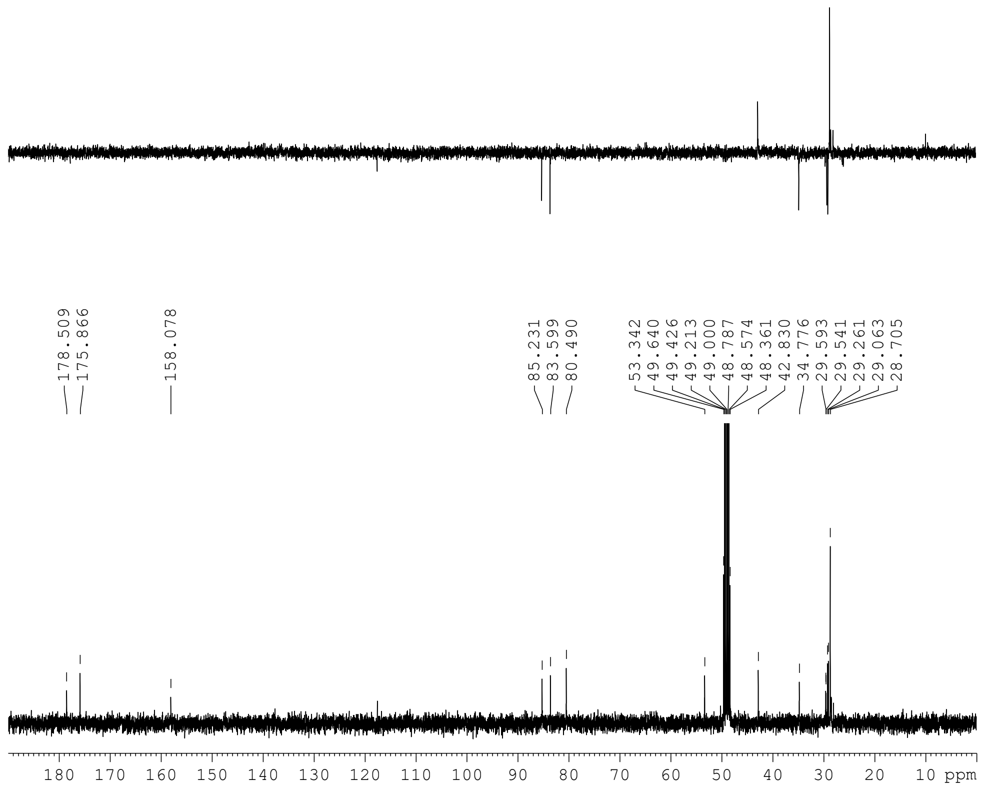

Figure 38. ^{13}C-NMR spectra of compound (2*R*,4*S*)-**14** (100 MHz, methanol-d_4).

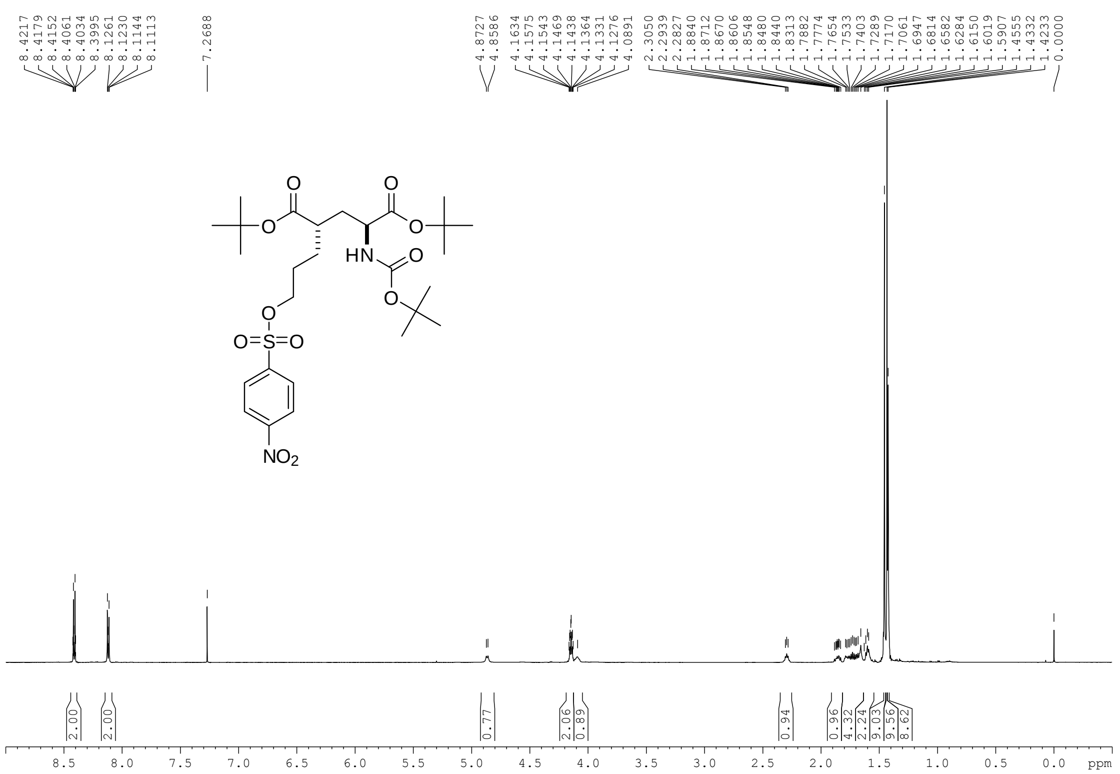

Figure 39. ^{1}H-NMR spectrum of compound (2*S*,4*S*)-**15** (600 MHz, $CDCl_3$).

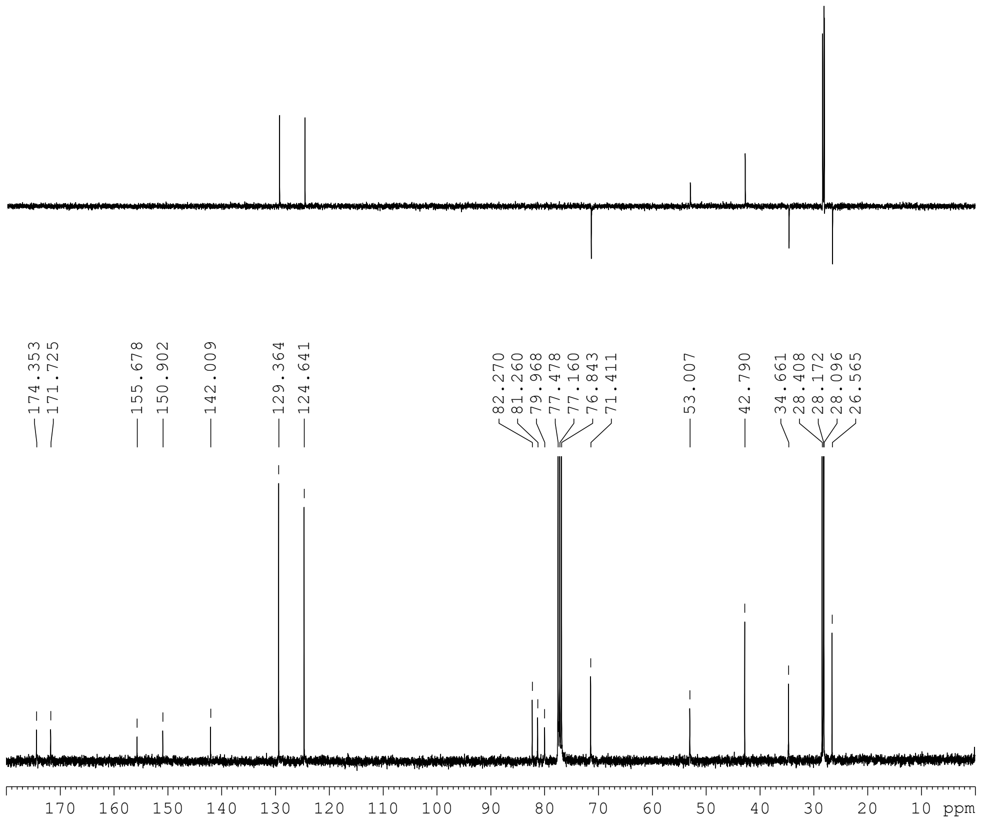

Figure 40. ^{13}C-NMR spectra of compound (2*S*,4*S*)-**15** (100 MHz, $CDCl_3$).

Equipment	*SHIMADZU*® : LC-6AD, SPD-20A
Column	Chiralcel OD (DAICEL) 250 ×4.6 mm<br>Guard column: Chiralcel OD 50 ×4.6 mm
Temperature	30°C
Mobile phase	1% 2-propanol in *n*-hexane
Flow rate	1.0 mL/min
T_R	(*S*)-**3**: 10.3 min<br>(*R*)-**3**: 7.7 min

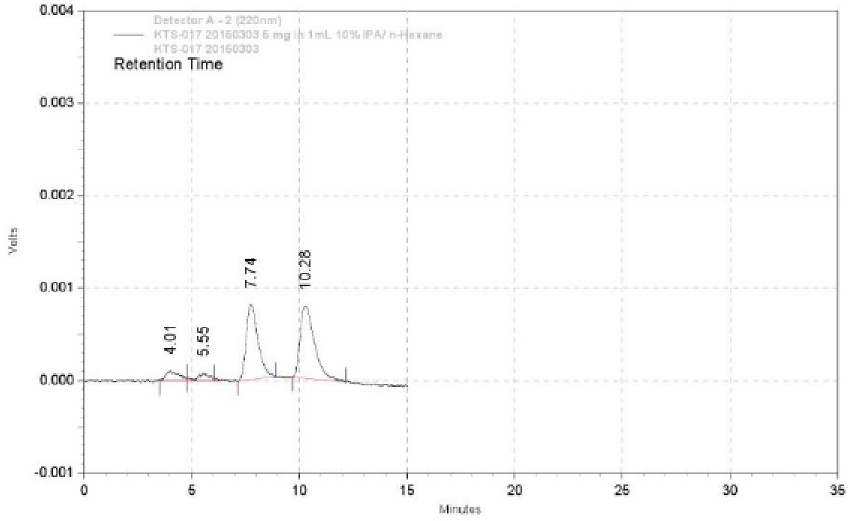

Detector A - 2
(220nm)

	Pk #	Retention Time	Area	Area Percent
1		4.01	4451	5.94
2		5.55	3214	4.29
3		7.74	30867	41.19
4		10.28	36408	48.58

Totals			
		74940	100.00

Figure 41. Chiral HPLC conditions and chromatogram of a mixture of (*S*)-**3** and (*R*)-**3**.

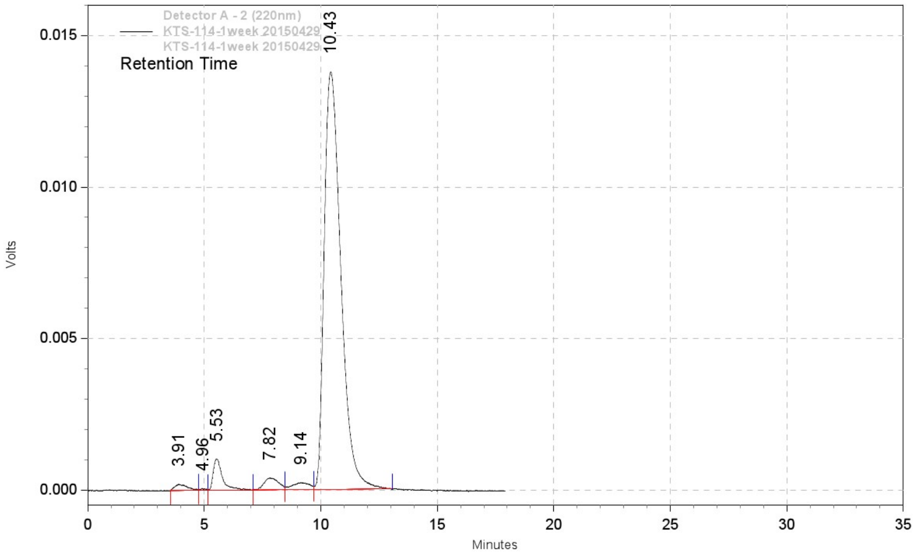

Detector A - 2 (220nm) Pk #	Retention Time	Area	Area Percent
1	3.91	8602	1.14
2	4.96	1287	0.17
3	5.53	29370	3.90
4	7.82	17536	2.33
5	9.14	12687	1.69
6	10.43	683141	90.77
Totals		752623	100.00

Figure 42. Chiral HPLC chromatogram of (*S*)-**3** in crude reaction mixture using $(Boc)_2O$.

Equipment	*SHIMADZU*® : LC-6AD, SPD-20A
Column	Chiralcel OD (DAICEL) 250 ×4.6 mm<br>Guard column: Chiralcel OD 50 ×4.6 mm
Temperature	18°C
Mobile phase	2% 2-propanol in *n*-hexane
Flow rate	1.0 mL/min
T_R	(2*S*,4*S*)-**6**: 22.1 min<br>(2*R*,4*R*)-**6**: 20.5 min

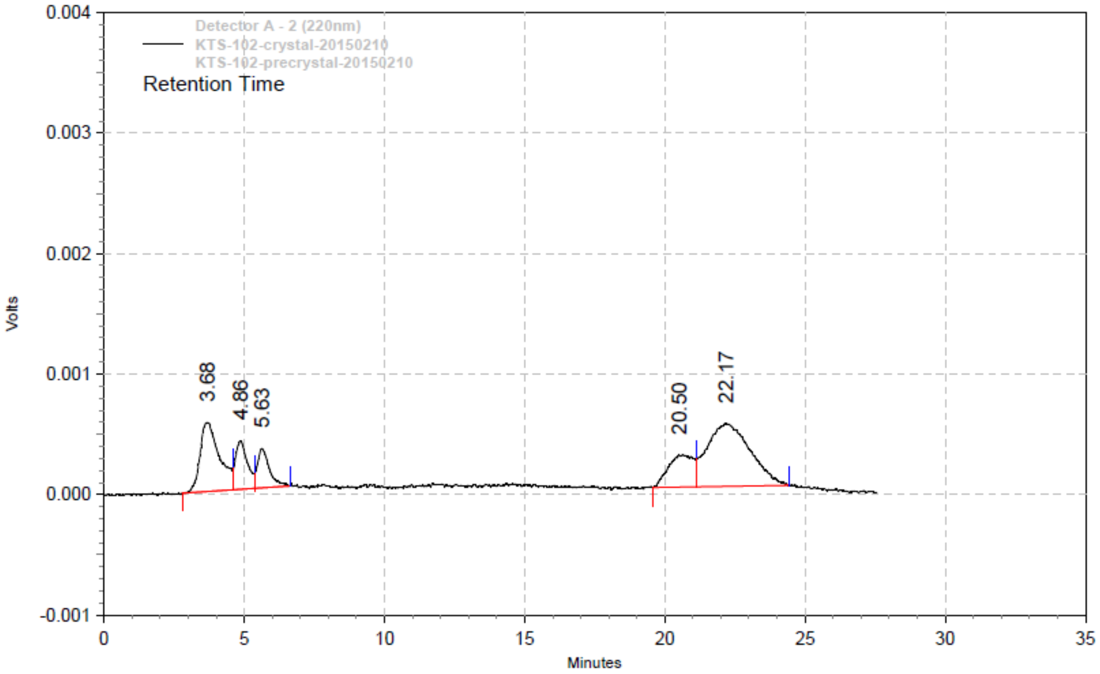

Figure 43. Chiral HPLC conditions and chromatogram of (2*S*,4*S*)-**6** and (2*R*,4*R*)-**6**.

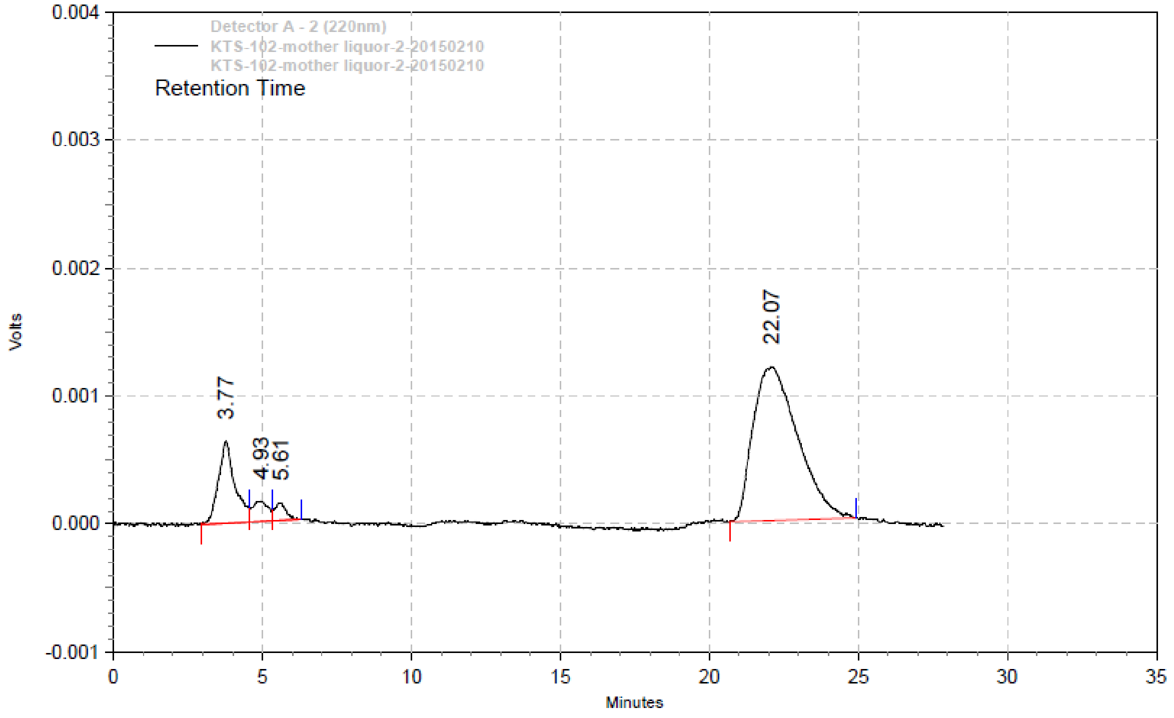

Figure 44. Chiral HPLC chromatogram of (2*S*,4*S*)-**6** after recrystallization.

Table 1. HPLC retention times for four stereoisomers of **1**.

	Retention time (t_R, min)		
compounds	RP-18[a]	Chiral HPLC[b] IPA	Chiral HPLC[b] MeCN
(2*S*,4*S*)-**1**	4.4	5.1	8.6
(2*R*,4*R*)-**1**		6.4	10.1
(2*S*,4*R*)-**1**	4.4	10.1	19.4
(2*R*,4*S*)-**1**		4.6	7.4

[a] Column: Phenomenex Luna C18, 5μ, 250×4.6 mm; mobile phase: 2 mM $CuSO_4$ aqueous solution.

[b] Column: Chirex 3126 (D)-penicillamine, 30×4.6 mm; mobile phase: 2 mM $CuSO_4$ aqueous solution/2-propanol = 85/15. [c] Column: Chirex 3126 (D)-penicillamine, 30×4.6 mm; mobile phase: 2 mM $CuSO_4$ aqueous solution/acetonitrile = 85/15.

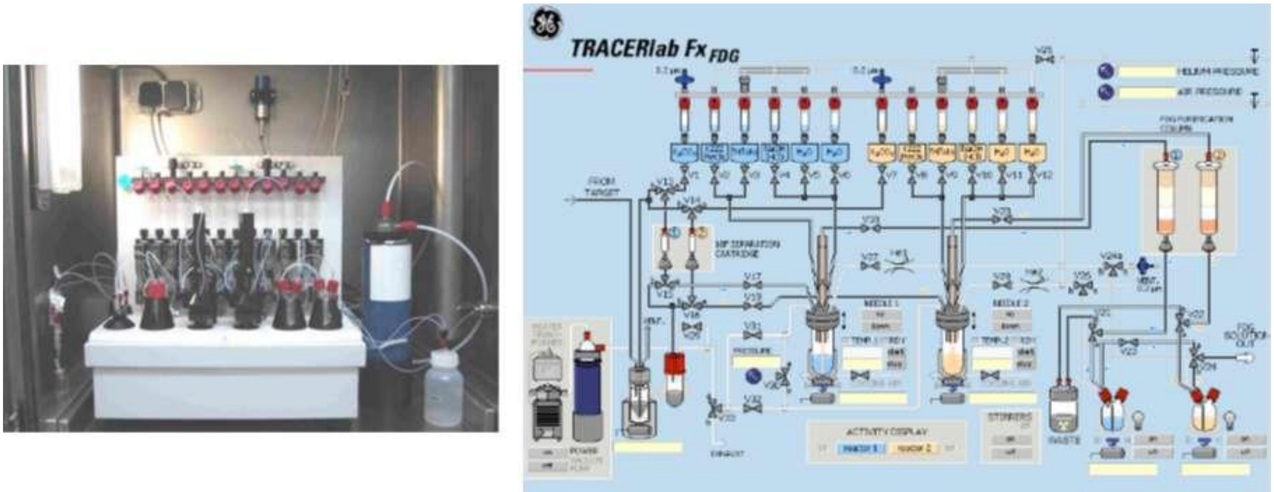

Figure 45. The GE TRACERlab FxFDG synthesizer at NTUH

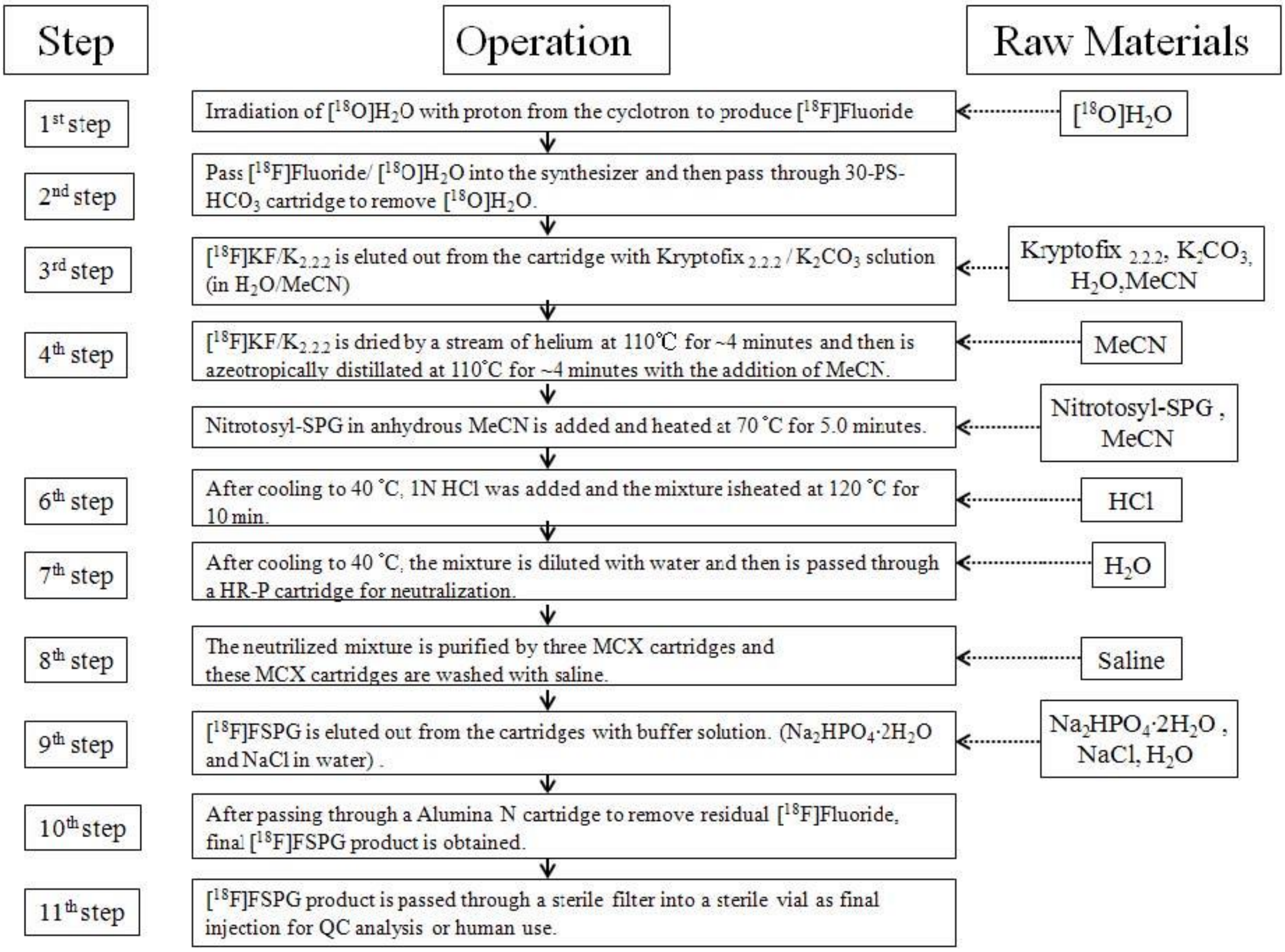

Figure 46. Flow chart of the process for radiosynthesis of $[^{18}F]$FSPG.

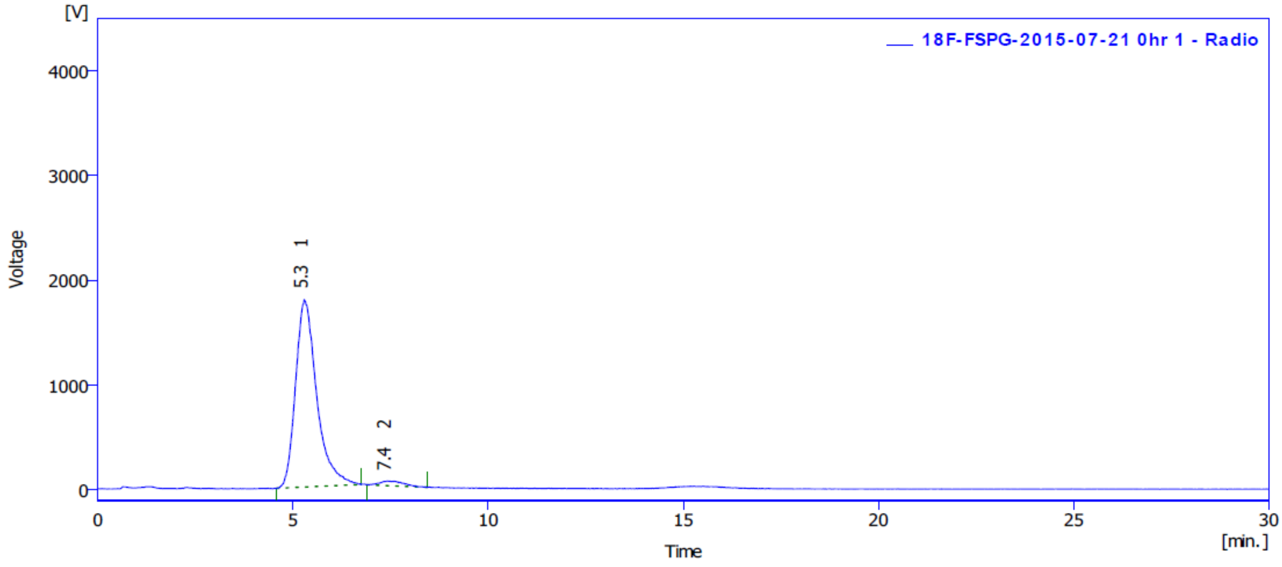

Result Table (Uncal - 18F-FSPG-2015-07-21 0hr 1 - Radio)

	Reten. Time [min]	Area [mV.s]	Height [mV]	Area [%]	Height [%]	W05 [min]	Symmetry/Tailing [-]	Compound Name
1	5.287	67424.152	1789.826	97.2	97.5	0.56	1.417	
2	7.417	1948.707	45.135	2.8	2.5	0.62	1.376	
	Total	69372.859	1834.961	100.0	100.0			

Figure 47. HPLC chromatogram of the [^{18}F]FSPG injection for clinical use. (Chirex 3126 column 30 x 4.6 mm, IPA/2 mM $CuSO_4$ = 10:90; at 20℃, 254 nm)

Table 2. Specifications for [^{18}F]FSPG injection.

Item	Specification
Appearance	A clear and colorless solution
pH	5~8
Sterility	Meet the requirement of injection
Bacterial endotoxins	≦ 17.5 IU/mL
Filter integrity test	> 40 psi
Radionuclide identity	> 99.5%
Half-life Measurement	110 ± 5 min
Radiochemical purity	≥ 90%
Enantiomeric Purity	≥ 90%
Diastereomers	≤ 5%
Residual Kryptofix-2.2.2	< 50 μg/mL
Residual solvent	EtOH ≤ 0.5%<br>MeCN ≤ 0.04%<br>acetone ≤ 0.5%
Residual fluoride	≤ 4%
