# Supplementary material for: Synthesis and analysis of 4-(3-fluoropropyl)-glutamic acid stereoisomers to determine the stereochemical purity of (4S)-4-(3-[18F]fluoropropyl)-L-glutamic acid ([18F]FSPG) for clinical use
Source: PLoS One. 2020 Dec 14;15(12):e0243831. doi: 10.1371/journal.pone.0243831 (PMC7735610; doi:10.1371/journal.pone.0243831)
Supplement: Attachment — Submitted filename: Point-by-point Response to the Comments.pdf [file pone.0243831.s005.pdf]

Synthesis and analysis of 4-(3-fluoropropyl)-glutamic acid stereoisomers to determine the stereochemical purity of (4S)-4-(3-[18F]fluoropropyl)-L-glutamic acid ([18F]FSPG) for clinical use

# Point-by-point Response to the Comments

**Journal Requirements:**

- The detail information about NMR experiments and data have been incorporated. Page 7 line 135-140; Page 8-17 line 167-399 and SI Figs 1-40 for individual compounds.

**Reviewer #1:**

1: The half life of 18F is 109.7 min, it is more appropriate to round its half life to 110 min, rather than109 min.

Response: Thanks! The half-life of 18F has been rounded to 110 min.

2: The separation resolution of four stereoisomers showed in Fig 7 is much better than in Fig 6. Their corresponding HPLC separation conditions, ranging from column length, solvent (IPA vs MeCN), temperature (23 centigrade vs 22 centigrade) to running time (15 min vs 25 min) are different. Compared with Fig 6, which one (or several) is the key point for good resolution? In Fig 6, column temperature proves to be crucial, but in Fig 7, it is hard to draw any conclusions for the improved resolution performance. Studies in Fig 7 will be more helpful only if it could offer some useful advices for people who may use your HPLC condition for similar separation, otherwise, it is confusing

3: More comparision studies need to be done to figure out what really matters for a good resolution. Temperature? Column length? Solvent or running time? The lack of such experiments devalues this paper.

Response: Thanks! The influences of temperatures and organic modifiers over the resolution and retention times in HPLC analysis have been carefully reinvestigated. The revised data, results and discussion are depicted on Page 29-32 line 636-691 and Figs 3-6. In summary: the $t_R$ and resolution of FSPG stereoisomers were sensitive to the temperature and organic modifiers: lower temperature resulted in longer $t_R$ and higher resolution; MeCN provided higher resolution and longer $t_R$ than IPA (Figs 3-6).

**Reviewer #2:**

1: The F-19 NMR spectra and optical rotations of compounds (2S, 4S)1, (2S, 4R)1, (2R, 4R)1 and (2R, 4R)1 should be added in this manuscript.

Response: Thanks! The F-19 NMR spectra and optical rotations of compounds **1** have been added. Page 8-9 line 167, 170-171, 178, 182, 187, 190-191, 197, and 201; SI Figs 3, 6, 9, and 12.

2：Why (S)-8 can be converted to (2S,4R) 11, but the related literature of FSPG can only get (2S,4S) derivatives, this reason should be explained in this manuscript.

Response: In Koglin's paper (ref 6), only (2*S*,4*S*)-derivatives were formed due to thermodynamic equilibrium of the acyclic enolate intermediates; whereas in Ezquerra's paper (ref 23), both (2*S*,4*R*)- and (2*S*,4*S*)-derivatives could be produced from L-pyroglutamate with low diastereoselectivity, which may be due to their similar energy of diastereomeric cyclic enolate intermediates. Thus, our finding in this study are consistent to the data in both references. The reasons are explained in Page 22 line 493-499 and Page 23 line 511-515 519-521

3: Since the purpose of this article is to promote the clinical production of [18F]FSPG, the detailed steps of radiolabeling in the article should be described, and the average yield of multiple high-dose labeling should be provided.

Response: Thanks! the detailed steps of radiolabeling is described in Page 17-18 line 403-429 and SI Fig 46. The average yield of multiple high-dose labeling is 4.4±2.4% (EOS, n=53) as shown in Page 32 line 704.

4：The physical and chemical properties of the final product [18F]FSPG should be provided.

Response: Thanks! The physical and chemical properties of the final product [18F]FSPG are provided on page 33 line 716-719 and SI Table 2.

**Reviewer #3:**

1: Figures 2, 3, and 5 could be presented as schemes as they contain reactions and not static structures.

Response: Thanks! Figures 2, 3, and 5 has been revised as Schemes 1-3.

2: Some yields are poor; while only small amounts of final product are needs as reference standards, the synthetic method would be more significant on its own if yields were optimized.

Response: Thanks! Since this manuscript is focused on the availability of all four stereoisomers for establishment of analytical methods, which has not been reported yet, further optimization of reaction conditions is an interesting topic and may be conducted in the future.

3: There is not a large amount of additional discussion in the discussion section, and it consists mainly as a restatement of the results section. As such, they could be combined into a results/discussion section to reduce redundancy.

Response: Thanks! The results and discussion sections have been combined into a results/discussion section as shown in Page 20-33, and the redundant contents have been deleted.

➢ Page 2 line 43 no comma after (2R,4R)-1,

Response: It has been revised as suggested!

➢ Page 2 line 54 specifications and was

Response: It has been revised as suggested!

➢ Page 8 line 163 residue was triturated with

Response: It has been revised as suggested!

➢ Page 8 line 164 77% no space

Response: It has been revised as suggested!

➢ Page 8 line 172 residue was triturated with

Response: It has been revised as suggested!

➢ Page 8 line 181 mL x 3 – x is different typeface, throughout the manuscript

Response: Here, the "×" is the multiplication sign to represent repeat three times, which is different from the lowercase "x"! Both "×" and "x" are Times New Roman typeface.

➢ Page 8 line 182 97% no space

Response: It has been revised as suggested!

➢ Page 9 line 198 g, no space

Response: It has been revised as suggested!

➢ Page 10 line 213 -78 °C space after number, throughout the manuscript

Response: In general, no space is needed between the numeral and the "°C" in Celsius temperature scale.

➢ Page 14 line 320 33% no space

Response: It has been revised as suggested!

➢ Page 16 line 365 mL x 2 space

Response: It has been revised as suggested!

➢ Page 17 line 400 10-60

Response: It has been revised as suggested!

- Page 20 line 463 BH3 subscript

Response: It has been revised as suggested!

- Page 20 line 464 C→rt, and throughout

Response: It has been revised as suggested!

- Page 24 line 546 0.05-0.07 use hyphens, not tildes, and throughout

Response: It has been revised as suggested!

- Page 25 line 568 mother liquor, and throughout

Response: It has been revised as suggested!

- Page 33 line 721 obtain permission

Response: It has been revised as suggested!
