# Supplementary material for: Synthesis and analysis of 4-(3-fluoropropyl)-glutamic acid stereoisomers to determine the stereochemical purity of (4S)-4-(3-[18F]fluoropropyl)-L-glutamic acid ([18F]FSPG) for clinical use
Source: PLoS One. 2020 Dec 14;15(12):e0243831. doi: 10.1371/journal.pone.0243831 (PMC7735610; doi:10.1371/journal.pone.0243831)
Supplement: Attachment — Submitted filename: Point-by-point Response to the Comments R2.pdf [file pone.0243831.s006.pdf]

Synthesis and analysis of 4-(3-fluoropropyl)-glutamic acid stereoisomers to determine the stereochemical purity of (4S)-4-(3-[18F]fluoropropyl)-L-glutamic acid ([18F]FSPG) for clinical use

Point-by-point Response to the Comments

Editor Comments:

1: Please add a statement about the use of CuSO4 at 2 mM. Why specifically at this concentration?

Response: Thanks! The $CuSO_4$ concentration was directly chosen based on the application example for the analysis of aspartic acid, which also possesses an acidic side-chain, in the column technical guide. A statement has been added to the text as suggested. Page 29, line 627-629.

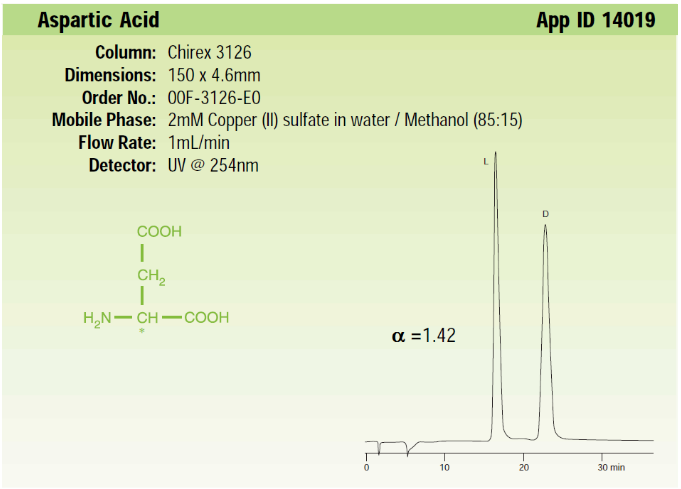

2: Most peaks in Figs S13, S15, S17 are difficult to observe, please provide an enlarge image to show those peaks with clear splitting patterns.

Response: Thanks! These figures have been enlarged as shown in Figs S13, S15, and S17.

3: 13C of (2S,4R)-11 contains too much impurities. Please update the spectrum using pure material.

Response: Thanks! The spectrum has been replaced as shown in Fig 24.
